# Gender differences in suicidal behavior in adolescents and young adults: systematic review and meta-analysis of longitudinal studies

**DOI:** 10.1007/s00038-018-1196-1

**Published:** 2019-01-12

**Authors:** Andrea Miranda-Mendizabal, Pere Castellví, Oleguer Parés-Badell, Itxaso Alayo, José Almenara, Iciar Alonso, Maria Jesús Blasco, Annabel Cebrià, Andrea Gabilondo, Margalida Gili, Carolina Lagares, José Antonio Piqueras, Tiscar Rodríguez-Jiménez, Jesús Rodríguez-Marín, Miquel Roca, Victoria Soto-Sanz, Gemma Vilagut, Jordi Alonso

**Affiliations:** 1 Health Services Research Group, IMIM-Institut Hospital del Mar d´Investigacions Mèdiques, PRBB Building. Doctor Aiguader 88, 08003 Barcelona, Spain; 2 Department of Health and Experimental Sciences, Pompeu Fabra University (UPF), Barcelona, Spain; 3 Department of Psychology, Jaen University, Jaén, Spain; 4 CIBER Epidemiología y Salud Pública (CIBERESP), Madrid, Spain; 5 Preventive Medicine Area and Public Health, University of Cádiz, Cádiz, Spain; 6 Morales Meseguer Hospital, Murcia, Spain; 7 Department of Mental Health, Corporació Sanitaria Parc Taulí, Sabadell, Spain; 8 Outpatient Mental Health Care Network, Osakidetza-Basque Health Service, San Sebastian, Spain; 9 Mental Health and Psychiatric Care Research Unit, BioDonosti Health Research Institute, San Sebastian, Spain; 10 Institut Universitari d’Investigació en Ciències de la Salut (IUNICS-IDISPA), University of Balearic Islands, Palma de Mallorca, Spain; 11 Network of Preventive Activities and Health Promotion, University of Balearic Islands, Palma de Mallorca, Spain; 12 Department of Statistics and Operative Research, University of Cádiz, Cádiz, Spain; 13 Department of Health Psychology, Miguel Hernandez University of Elche, Elche, Spain

**Keywords:** Gender, Suicide, Suicide attempt, Adolescents, Young adults, Risk factors

## Abstract

**Objectives:**

To assess the association between gender and suicide attempt/death and identify gender-specific risk/protective factors in adolescents/young adults.

**Methods:**

Systematic review (5 databases until January 2017). Population-based longitudinal studies considering non-clinical populations, aged 12–26 years, assessing associations between gender and suicide attempts/death, or evaluating their gender risk/protective factors, were included. Random effect meta-analyses were performed.

**Results:**

Sixty-seven studies were included. Females presented higher risk of suicide attempt (OR 1.96, 95% CI 1.54–2.50), and males for suicide death (HR 2.50, 95% CI 1.8–3.6). Common risk factors of suicidal behaviors for both genders are previous mental or substance abuse disorder and exposure to interpersonal violence. Female-specific risk factors for suicide attempts are eating disorder, posttraumatic stress disorder, bipolar disorder, being victim of dating violence, depressive symptoms, interpersonal problems and previous abortion. Male-specific risk factors for suicide attempt are disruptive behavior/conduct problems, hopelessness, parental separation/divorce, friend’s suicidal behavior, and access to means. Male-specific risk factors for suicide death are drug abuse, externalizing disorders, and access to means. For females, no risk factors for suicide death were studied.

**Conclusions:**

More evidence about female-specific risk/protective factors of suicide death, for adolescent/young adults, is needed.

**Electronic supplementary material:**

The online version of this article (10.1007/s00038-018-1196-1) contains supplementary material, which is available to authorized users.

## Introduction

Suicide is a very serious public health concern. In 2016, there were an estimated 793,000 suicide deaths worldwide, representing an annual global age-standardized suicide rate of 10.5 per 100,000 population. Globally, it is the second leading cause of death among persons aged 15–29 years (World Health Organization 2016). In adolescents and young adults, suicide rates are 2–4 times higher in males than in females, while suicide attempts are 3–9 times more common in females (Wunderlich et al. 2001; Eaton et al. 2012). In developed countries, suicide mortality has been estimated to be 2–3 times higher in young males than females (Wasserman et al. 2005).

Within the context of suicide research, gender differences in suicidal behavior rates are known as the “Gender Paradox” (Canetto and Sakinofsky 1998). In adolescents and young adults, this paradox changes according to age (Canetto 2008; Rhodes et al. 2014a). Female suicide attempt rates increase with age, peaking in mid-adolescence (Lewinsohn et al. 2001; Boeninger et al. 2010; Thompson and Light 2011), whereas male suicide rates increase until early adulthood (World Health Organization 2014). Previous suicide attempts are one of the strongest predictors of suicide death (Kokkevi et al. 2012), especially among females. Gender differences in suicidal behavior may be explained by differences in emotional and behavioral problems (Kaess et al. 2011). The higher rates of suicide deaths among male youths may be associated with a higher prevalence of externalizing disorders (e.g., conduct disorder, substance abuse disorder, deviant behavior) (Mergl et al. 2015) and a preference for highly lethal methods (Värnik et al. 2008). In contrast, females are more prone to show internalizing disorders (e.g., anxiety, mood disorders) (Fergusson et al. 1993). These disorders may mediate the association with suicidal thoughts and behaviors (Peter and Roberts 2010; Mars et al. 2014).

To the best of our knowledge, no previous meta-analysis has assessed the association between gender and suicidal behaviors, or gender-specific determinants, in adolescents and young adults. Accurately identifying gender-specific risk and protective factors for suicidal behaviors is important to improve knowledge and to develop more effective suicide prevention programs. Therefore, we undertook a systematic review of the literature aiming to: (1) assess the magnitude of association between gender and suicide attempts and death; and (2) to identify gender-specific risk and protective factors of suicide attempts and death in adolescents and young adults.

## Methods

This article is based on a broad, comprehensive systematic review of the risk and protective factors of suicidal behaviors in adolescents and young adults aged 12–26 years. The recommendations of the MOOSE guidelines for systematic reviews were followed (Table S1) (Stroup et al. 2000). The original search protocol was registered at PROSPERO. More information about the search strategy and selection criteria is provided in Text S1 (available online).

For this article, specific selection criteria were applied, including: (1) cohort studies assessing the association between gender and suicide attempts or death; and (2) cohort or case–control studies evaluating risk or protective factors for suicide attempts or death stratified by sex. For the assessment of gender with suicidal behaviors, case–control studies were excluded because the subjects were matched by sex, which may lead to underestimation of risk. To assess suicide attempts, we considered females as the subpopulation at risk, with males as the comparison group; for suicide death, males were the subpopulation at risk (World Health Organization 2014). An exhaustive peer review process was used to classify risk and protective factors according to their definition in the primary studies, a previous exhaustive review of the literature (Evans et al. 2004) and the World Health Organization’s socio-ecological model (World Health Organization 2014). The principal categories were as follows: sociodemographic and educational, individual negative life events and family adversity, psychiatric/psychological factors, personal factors and community factors.

A Cochrane Collaboration data collection form was adapted for data extraction (Higgins and Green 2008). Data were extracted by two reviewers, and a third assessed whether the information was entered properly and attempted to complete any missing data. If there were discrepancies, consensus was established among reviewers. The following data were extracted from each article: (1) sample size, (2) prevalence of females and males, (3) age range, (4) mean age, (5) country of recruitment, (6) study design, (7) suicide outcome, (8) type of sample recruited, (9) adjustment variables, and (10) ethics committee approval. For cohort studies, additional data extraction included: (1) weeks of follow-up, (2) number of suicide attempts or suicide deaths during follow-up, and (3) attrition rates. Information about sex-stratified risk and protective factors was obtained as follows: odds ratio (OR) and 95% confidence intervals (95% CI) or beta coefficients and standard errors (SE). Multivariate analyses were selected over bivariate analyses. If there were multiple publications on the same sample and factors, the results of the largest sample and longest follow-up were selected for the analyses.

### Quality assessment

The Newcastle–Ottawa scale (NOS) was used to assess the quality of non-randomized studies (Wells et al. 2013), including: (1) selection of study groups, (2) comparability between groups, and (3) exposure in case–control studies or outcome in cohort studies. The NOS includes eight questions (four in selection, one in comparability, and three in exposure or outcome) with various response options; the response indicating the highest quality is assigned 1 point. One point can be granted for each question within the selection and exposure or outcome categories. For comparability, a maximum of 2 points can be given. The highest quality studies may receive a maximum of 9 points.

### Data synthesis

Meta-analyses were performed when there were a minimum of two studies with usable data; random effect methods were used. Heterogeneity was assessed by visual inspection of forest plots, Galbraith plots, a Chi-square test to calculate *p* value, and the Higgins test (*I*^2^), which describes the percentage of observed heterogeneity that would not be expected by chance. Heterogeneity was considered to be significant when *p* was < 0.10, and was classified as low (< 30%), moderate (30%–50%), and severe (> 50%) (Higgins and Thompson 2002). Small study effects (including publication bias) were assessed through visual inspection of funnel plots and the Egger test. Sensitivity analyses were only conducted for the analysis of gender as a risk factor, according to two criteria: (1) publication year (studies published before the year 2000) and (2) NOS scale < 6 points. Meta-analyses assessing the effects of risk and protective factors on suicide attempts and death were carried out. Due to the large number of figures, those not presented in this article are available upon request. STATA software version 13 was used.

## Results

**Fig. 1:**
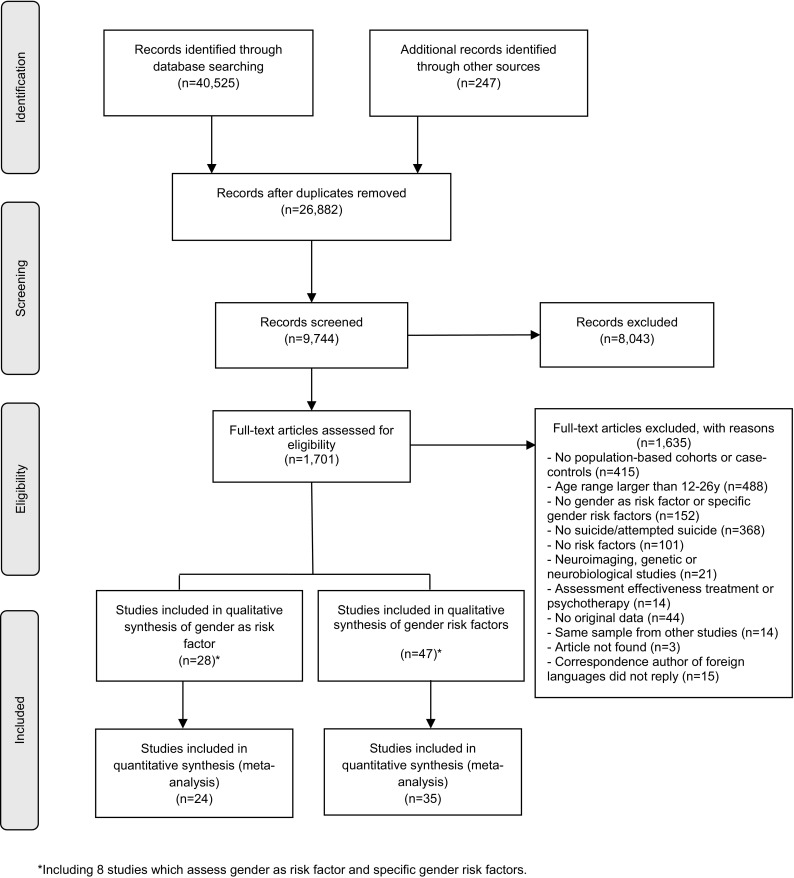
Modified version of PRISMA diagram of the included studies in the systematic review of gender differences in suicidal behavior in adolescents and young adults (covered up until January 2017)

Of 26,882 potentially suitable articles, we identified 1701 full-text articles for eligibility. Of these, 1635 were excluded. Reasons for exclusion are detailed in Fig. 1. A total of 77 articles or publications were included, representing 67 distinct studies. Ten articles were excluded from the analyses as they reported results from the same samples but with shorter follow-up periods and without providing any additional information. The references of all included articles are provided in supplementary Text S2. Nineteen studies assessed the association between gender and suicide attempts; one assessed the association between gender and suicide death; 39 assessed sex-specific risk and/or protective factors for the outcomes; and eight assessed both gender and sex-specific risk and/or protective factors. Results are presented separately for suicide attempts and suicide death.

### Quality of reviewed studies

No relevant differences between the included studies were observed in the selection domain. For comparability, 39 studies achieved two points. The lowest scores were found in exposure or outcome domains: Only 15 studies achieved 1 point in the question about the ascertainment of the outcome or exposure, because most studies included self-reported data without confirmatory records; 34 studies received 1 point because the length of follow-up was ≥ 6 years; and 25 studies received 0 points for adequacy of follow-up (attrition rates were > 25%). More information is detailed in Table S2 (available online).

### Gender as a risk factor for suicidal behavior

#### Suicide attempts

Articles were published between 1995 and 2017, including samples predominantly from the USA (*n* = 13) and Canada (*n* = 4). Participation rates ranged from 3% to almost 98%. A summary of the most relevant characteristics of the included studies is presented in Table 1.

**Table 1:** Characteristics of the included studies in the systematic review of gender differences in suicidal behavior in adolescents and young adults (covered up until January 2017)

Author (study)	Country	Follow-up	Sample at baseline (% of women)	Sample at the end of follow-up (% of attrition)	Percentage of suicide attempts or deaths during the follow-up	Population	Age range (years)	Mean age (standard deviation)	Meta-analysis (gender, risk and protective factors or both)
*Studies assessing suicide attempt*									
Cohort studies									
Kaplan and Pokonny (1976)	USA	3 years	7618	3148 (58.7)	NI	High school	NI	NI	None
Reinherz et al. (1995)	USA	14 years	404	385 (4.7)	4.2	High school	18	17.9	Gender
Silverman et al. (1996)	USA	17 years	777 (49.9)	375 (51.7)	2.7	General	15–21	NI	Risk and protective factors
McKeown et al. (1998)	USA	2 years	359 (56)	359	1.7	High school	NI	NI	Gender
Wichstrom (2000)	Norway	2 years	11,918 (41.1)	9679 (18.8)	8.2	High school	14–22	NI	Both
Borowsky et al. (2001)	USA	1 year	20,745 (52)	13,110 (37)	36	Students	13–19	16	None
Lewinsohn et al. (2001)	USA	8 years	1709 (57)	941 (45)	13,2	Students	24	24	Risk and protective factors
Sourander et al. (2001, 2009)	Finland	8 years	6017	580 (90.4)	0.46	General	16	16.0 (0.5)	Both
Fergusson et al. (2003) (Christchurch)	New Zealand	21 years	1265 (49.8)	1063 (15.9)	7.3	General	21–25	NI	Gender
Bearman et al. (2004)	USA	1 year	20,745	13,465 (35)	NI	High school	NI	NI	Risk and protective factors
Ialongo et al. (2004)	USA	11 years	1197 (56)	747 (38)	44.1	Students	19–20	NI	Risk and protective factors
D´Augelli et al. (2005)	USA	2 years	528	361 (31.6)	17	LGB	15–19	NI	Both
Feigelman et al. (2006), Thompson et al. (2007), Exner-Cortens et al. (2013), Van Dulmen et al. (2013), Abrutyn et al. (2014), Turanovic and Pratt (2015) (Add Health)	USA	14 years	20,745	13,110 (36.8)	3.6	High school	NI	NI	Both
Kidd et al. (2006)	USA	1 year	12,105	9142 (24.5)	NI	High school	12–17	16.0	Gender
Rodríguez-Cano et al. (2006)	Spain	2 years	1776 (49.9)	1076 (39.4)	3.8	High school	13–15	NI	Gender
Ackard et al. (2007)	USA	5 years	3074	1710 (44.4)	3.9 females dating violence; 4.4 males dating violence	Students	NI	20.4 (0.8)	Risk and protective factors
Brezo et al. (2007, 2008) (Quebec)	Canada	22 years	3017 (47.2)	1776 (41.1)	9.3 any; 1.8 repeated	Students	19–24	21.4	Gender
Crow et al. (2008)	USA	5 years	3672	2516 (22.6)	8.7 females; 3.5 males	General	NI	17.2 high school; 20.4 young adults	Risk and protective factors
Dupéré et al. (2008)	Canada	10 years	4951	2776 (43.9)	NI	General	12–19	NI	Gender
Lambert et al. (2008)	USA	3 years	678 (46.5)	473 (30.2)	NI	Students	13–14	13.8 (0.3)	None
Nrugham et al. (2008, 2015)	Norway	6 years	2464 (50.8)	265 (89.2)	1.50	Students	18–21	20 (0.6)	Risk and protective factors
Wong et al. (2008)	China	12 months	1747 (34)	1099 (37.1)	NI	Students	12–18	14.5	Both
Wilcox et al. (2009)	USA	17 years	2311 (50.2)	1570 (47.2)	2.38	Students	20–23	21	Both
Batty et al. (2010)	Sweden	24 years	1,379,531	1,133,019 (17.9)	NI	General	16–25	18	None
Tracey et al. (2010) (NLSCY)	Canada	5 years	25,781	2499 (90.3)	45.9	General	15–18	NI	Both
Roberts et al. (2010)	USA	1 year	4500	3134 (30.3)	0.84	General	11–17	NI	Gender
Klomek et al. (2011)	USA	4 years	2342	342	NI	High school	13–18	14.8 (1.2)	Risk and protective factors
Young et al. (2011)	UK	8 years	2586 (50.4)	1860 (28.1)	6.1	High school	15	NI	Gender
Fried et al. (2012)	USA	2 years	27,000	1728 (93.6)	0.5	Students	16–18	16.7 (0.6)	Gender
Guan et al. (2012)	USA	2.5 years	712 (54.8)	399 (44)	5.2	High school	NI	NI	Gender
Hurtig et al. (2012)	Finland	16 years	9215	273 (97.1)	0.086	General	15–18	NI	Gender
Nkansah-Amankra et al. (2012)	USA	14 years	20,715	9412 (54.6)	1.9	General	18–26	NI	Risk and protective factors
Wanner et al. (2012)	Canada	22 years	3017 (47.2)	1776 (41.1)	2.3	Students	19–24	21.4	Risk and protective factors
Winterrowd and Canetto (2013)	USA	3 years	295 (59)	253 (14.2)	9	General	19–23	19.5 (1.1)	None
Chang et al. (2014) (ALSPAC)	UK	17 years	14,062	3560 (74.6)	1.61	General	16–17	16.8 (2.9)	None
Chuan-Yu et al. (2014)	China	1 year	9393	7313 (44)	NI	General	15–24	NI	Risk and protective factors
Conner et al. (2014)	USA	5 years	NI	418	4.54	General	12–19	NI	Gender
Luntano et al. (2014)	Finland	16 years	6017	5416 (6.8)	0.88	General	NI	NI	Risk and protective factors
Mars et al. (2014)	UK	16 years	14,062	4799 (65.9)	3.5	General	16–17	NI	Gender
Miranda et al. (2014)	USA	6 years	1729	506 (70.7)	2.4	Students	12–20	15.6 (1.4)	Gender
Soller et al. (2014)	USA	12 years	5316	4459 (16.1)	4.72	Students	NI	NI	Risk and protective factors
Swanson et al. (2014)	USA	10 years	228	199 (12.7)	NI	Students/females	16–22	19.6	Risk and protective factors
Scott et al. (2015)	USA	16 years	2450	1950 (20.4)	1.47	General/females	17–21	NI	Risk and protective factors
You et al. (2015)	China	1 year	5423 (53)	3600 (34)	2,9	Students	12–18	14.63 (1.3)	Both
Conway et al. (2016)	Denmark	9 months	99 (88.9)	85 (14.1)	14.1	General	NI	16.3 (1.6)	None
Meza et al. (2016)	USA	10 years	228 (100)	216 (5)	NI	General	17–24	19.6	None
Mok et al. (2016)	Denmark	30 years	1,743,525 (48.7)	1,743,525	2.6	General	NI	21.6	None
Hishinuma et al. (2017) (HHSHS study)	USA	5 years	2133	2083 (2.34)	2.7 females; 1.6 males	Native Hawaiians/non–Hawaiians	14–17	NI	None
Case–control studies									
King et al. (1990)	USA	*a*	19 cases vs. 21 controls (100)	*a*	*a*	General/Women	NI	14.9 (1.2)	None
Rotheram-Borus and Shrout (1990)	USA	*a*	77 cases vs. 63 controls (100)	*a*	*a*	Primary health care	12–17	14.7	Risk and protective factors
Garnefski et al. (1992)	The Netherlands	*a*	285 cases vs. 285 controls (64.9)	*a*	*a*	Students	13–20	16	Risk and protective factors
Adams et al. (1994)	USA	*a*	91 cases vs. 155 controls	*a*	*a*	High school	12–17	NI	None
Beautrais et al. (1998)	New Zealand	*a*	129 cases vs. 153 controls	*a*	*a*	General	18–24	19.4 (3.0) cases; 21.4 (1.6) controls	Risk and protective factors
Lyon et al. (2000)	USA	*a*	38 cases vs. 76 controls (18.4)	*a*	*a*	Primary care	12–17	14.8	Risk and protective factors
Ikeda et al. (2001)	USA	*a*	153 cases (37) 513 controls (40)	*a*	*a*	General	13–24	NI	Risk and protective factors
Donald et al. (2005)	Australia	*a*	95 cases vs. 380 controls (48)	*a*	*a*	General	18–24	NI	Risk and protective factors
Bilgin et al. (2007)	Turkey	*a*	52 cases vs. 52 controls (100)	*a*	*a*	High school	14–18	16	None
Freitas et al. (2008)	Brazil	*a*	110 cases vs. 110 controls (100)	*a*	20 cases, 6.3 controls (prevalence)	Primary health care	14–18	NI	Risk and protective factors
Christiansen et al. (2011, 2012)	Denmark	*a*	3465 cases vs. 69,300 controls (78.7)	*a*	4.8	General	16–22	16.8 (2.3) females; 17.8 (2.4) males	Risk and protective factors
*Studies assessing suicide death*									
Cohort studies									
Finkelstein et al. (2015)	Canada	12 years	1,044,405	1,043,958 (0.042)	0.039	General	17–26	NI	Gender
Feigelman et al. (2016) (Add Health study)	USA	7 years	20,771	10,122	21	General/men	20–26	NI	None
Weiser et al. (2016)	Israel	16 years	988,847	634,655 (35.8)	0.07	General/men	16–17	16.9 (0.5)	None
Case–control studies									
Salk et al. (1985)	USA	*a*	52 cases vs. 104 controls (17.3)	*a*	*a*	General	12–20	NI	None
Brent et al. (1993, 1999)	USA	*a*	67 cases vs. 67 controls; 140 cases vs. 131 controls (22)	*a*	*a*	General	13–19	14	Risk and protective factors
Shaffer et al. (1996)	USA	*a*	120 cases vs. 147 controls	*a*	*a*	General	20	15.9 females; 16.9 males	Risk and protective factors
Cheng et al. (2014)	China	*a*	500 cases vs. 15,000 controls (47.6)	*a*	*a*	General	15-19	NI	Risk and protective factors
*Studies assessing suicide death and suicide attempt*									
Case–control studies									
Ostry et al. (2007)	Canada	*a*	827	*a*	*a*	General	NI	NI	Risk and protective factors

*NI* no information, *a* not applicable

**Fig. 2:**
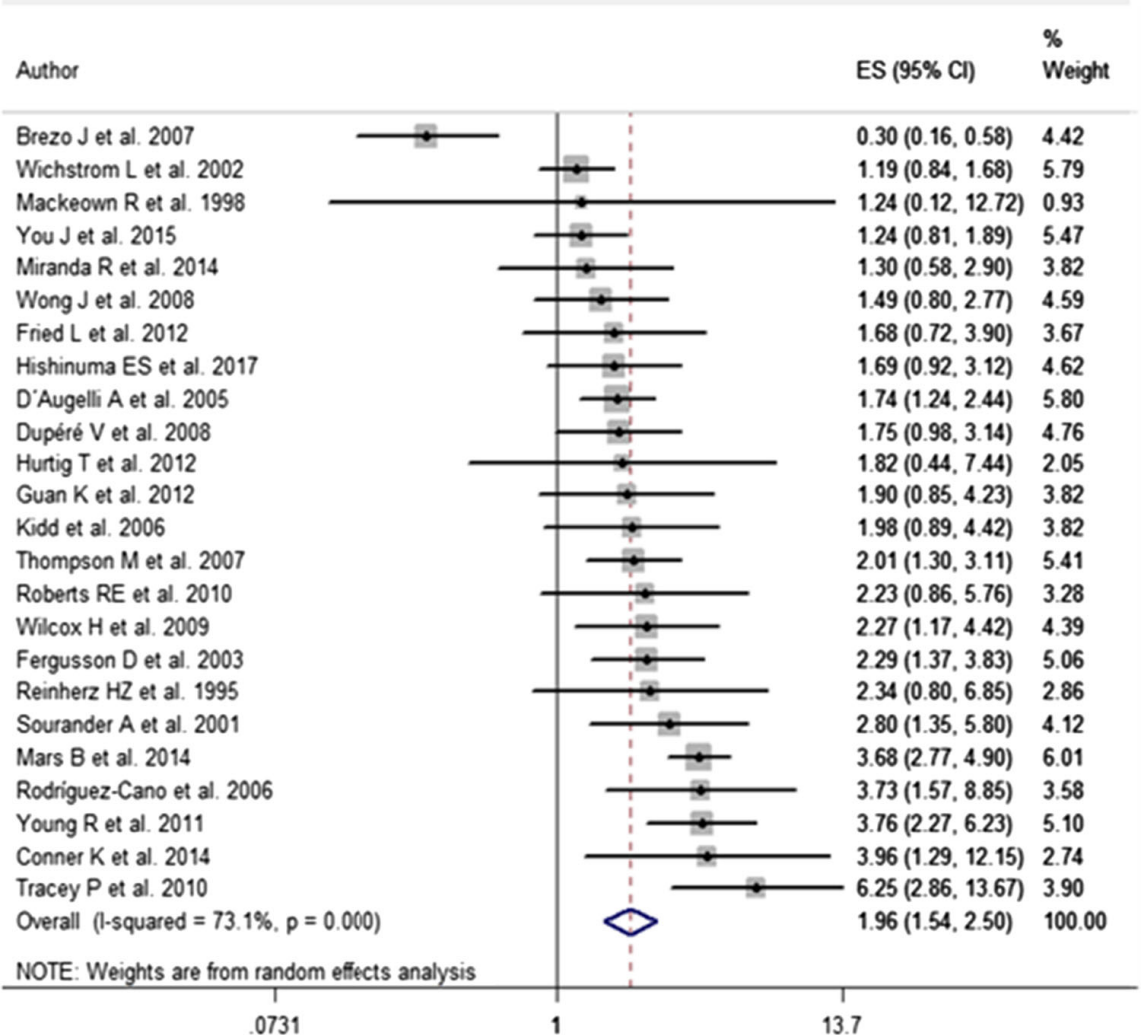
Forest plot of being female as risk factor of suicide attempt—results of the systematic review of gender differences in suicidal behavior in adolescents and young adults (covered up until January 2017)

Of the 27 studies assessing the association between gender and suicide attempts, 24 were included in the meta-analysis. Three studies were excluded because the data were either non-extractable or did not allow comparisons. Compared with males, females showed a significantly higher pooled risk of suicide attempts (OR 1.96, 95% CI 1.54–2.50), although high heterogeneity was observed (*I*^2^= 73.1%; *p* < 0.001) (Fig. 2). The funnel plot appeared asymmetric, but the Egger test did not suggest the existence of any publication bias (*p* = 0.847). After sensitivity analyses, according to publication year and quality score, no significant changes were seen.

#### Suicide death

One cohort study explored the association between gender and suicide death, including a sample of 1,043,958 subjects. A total of 20,471 surviving adolescents (median age 16 years; IQR 15–18), attended in the emergency department for a first self-poisoning episode, were followed from the date of discharge until death or the end of the study, whichever occurred first. Fifty matched population-based reference individuals were selected for each surviving adolescent (*n* = 1,023,487). After a median follow-up time of 7.2 years (IQR 4.2–9.7), the results showed that 126 individuals (0.6%) in the self-poisoning group and 286 (0.03%) in the reference group died by suicide. After a self-poisoning episode, death from suicide was more than twice as likely among males compared with females (HR 2.5, 95% CI 1.8–3.6) (Finkelstein et al. 2015).

### Specific risk factors for suicidal behavior stratified by gender

A full summary of results for all risk and protective factors assessed is detailed in Table 2.

**Table 2:** Meta-analysis results of gender risk and protective factors of suicidal behavior among adolescents and young adults—results of the systematic review of gender differences in suicidal behavior in adolescents and young adults (covered up until January 2017)

Factor(s)	Female					Male				
	Studies (*n*)	Samples (*n*)	OR	95% CI	*I* ^2^	Studies (*n*)	Samples (*n*)	OR	95% CI	*I* ^2^
*Suicide attempt*										
Sociodemographic and educational										
Academic factors	3	3	0.94	0.80–1.11	0	3	3	1.41	0.72–2.74	79.8
Low socioeconomic status	2	3	1.52	0.89–2.58	99.1	2	3	1.65	0.83–3.27	98.1
Parental education	2	2	1.78	0.91–3.47	0	2	2	0.99	0.51–1.92	0
Race/ethnicity	3	3	0.98	0.68–1.41	0	2	2	0.93	0.53–1.61	0
Individual negative life events and family adversity										
Any negative life event ^a^	6	6	1.31	0.93–1.86	94.7	6	6	1.22	0.98–1.51	75.8
Bullying	**1**	**1**	**6.30**	**1.53–25.90**	**NA**	**1**	**1**	**3.8**	**1.01–14.30**	**NA**
Childhood maltreatment	**3**	**5**	**3.77**	**2.13–6.68**	**69.6**	**3**	**4**	**2.76**	**1.20–6.36**	**72.8**
Community violence	**3**	**3**	**1.68**	**1.42–1.99**	**0**	**2**	**2**	**1.83**	**1.48–2.26**	**0**
Conflicts with partner	2	2	1.2	0.87–1.65	67.6	1	1	1.05	0.52–2.13	NA
Dating violence	**2**	**3**	**2.19**	**1.29–3.71**	**0**	3	3	1.45	0.54–3.86	32.3
Parental separation or divorce	7	8	1.07	0.88–1.29	27.2	**7**	**8**	**1.56**	**1.01–2.41**	**73.4**
Family history of mental disorders and abuse	**2**	**3**	**2.27**	**1.78–2.89**	**18.8**	**3**	**6**	**2.63**	**1.99–3.47**	**98.6**
Family previous suicidal behavior	2	3	2.10	0.97–4.58	93.2	**3**	**4**	**2.84**	**1.87–4.33**	**42.4**
Interpersonal difficulties	**2**	**3**	**1.13**	**1.03–1.24**	**0**	1	2	1.04	0.90–1.21	0
Psychiatric and psychological										
Psychiatric										
ADHD	3	4	0.79	0.19–3.21	78.8	1	1	4.50	0.96–21.20	NA
Alcohol abuse disorder	**2**	**2**	**2.69**	**1.32–5.50**	**0**	**2**	**2**	**2.14**	**1.09–4.20**	**0**
Alcohol use	3	3	1.16	0.83–1.62	78.0	3	3	1.10	0.94–1.27	6.3
Anxiety disorder	**3**	**4**	**2.03**	**1.77–2.33**	**0**	**3**	**5**	**3.79**	**2.05–7.01**	**91.8**
Any mental disorder or abuse	**10**	**36**	**3.37**	**2.52–4.51**	**88.4**	**6**	**27**	**4.23**	**3.28–5.47**	**0.8**
Bipolar disorder	**2**	**2**	**1.43**	**1.20–1.70**	**0**	No data				
Conduct disorder	1	1	2.31	0.50–10.65	NA	1	1	0.80	0.10–6.53	NA
Drug abuse disorder	**3**	**6**	**4.44**	**2.51–7.83**	**72.2**	**2**	**5**	**3.11**	**2.01–4.84**	**0**
Drugs use	3	3	3.2	0.68–14.95	78.9	3	3	3.03	0.64–14.32	87.7
Eating disorder	**1**	**2**	**5.27**	**2.04–13.60**	**0**	No data				
Gambling disorder	1	1	4.13	0.54–31.85	NA	1	1	1.01	0.14–7.35	NA
Major depressive disorder	**4**	**5**	**4.49**	**2.18–9.23**	**78.4**	**3**	**4**	**6.07**	**1.74–21.20**	**83.6**
NSSI	2	2	2.03	0.52–7.89	88.2	1	1	1.00	0.92–1.09	NA
Personality disorder	**1**	**2**	**7.89**	**3.81–16.35**	**0**	**2**	**2**	**5.13**	**2.63–10.01**	**0**
PTSD	**2**	**2**	**2.96**	**1.32–6.62**	**38.6**	1	1	3.57	0.58–22.16	NA
Previous suicidal ideation	**4**	**4**	**4.39**	**2.31–8.34**	**77.5**	**4**	**4**	**3.97**	**1.40–11.24**	**84.5**
Previous suicide attempts	**5**	**7**	**6.96**	**3.75–12.91**	**58.2**	**1**	**2**	**31.33**	**9.36–104.88**	**0**
Psychological										
Aggressiveness	No data					1	1	1.15	0.67–1.98	NA
Anxiety symptoms	No data					1	1	0.64	0.40–1.03	NA
Depressive symptoms	**10**	**10**	**1.15**	**1.04–1.28**	**66.9**	6	6	1.26	0.98–1.62	61.5
Disruptiveness	3	5	2.54	0.67–9.60	80.7	**2**	**3**	**8.78**	**2.77–27.84**	**75.6**
Hopelessness	3	3	1.55	0.71–3.42	69.4	**3**	**3**	**1.74**	**1.04–2.94**	**0**
Low self-esteem	4	4	1.46	0.78–2.74	87.0	4	4	1.22	0.95–1.57	0
Self-concept	3	4	1.35	0.92–1.96	50.0	3	4	1.51	0.93–2.44	57.2
Personal										
Abortion	**1**	**2**	**1.3**	**1.09–1.55**	0	NA				
Any medical condition	3	5	1.01	0.98–1.04	0	2	3	1.21	0.84–1.72	43.7
Body mass index	2	2	1.01	0.98–1.05	0	2	2	0.98	0.93–1.03	0
Dating	1	5	1.03	0.95–1.11	35.0	1	5	0.97	0.82–1.14	42.6
Eating behaviors	3	5	1.26	0.91–1.75	72.8	3	4	1.06	0.95–1.19	0
Pregnancy in females	2	2	1.65	0.36–7.56	82.3	NA				
Religiosity	2	3	0.87	0.67–1.12	0	2	3	1.12	0.76–1.63	0
Somatic symptoms	2	3	1.48	0.82–2.68	29.7	1	2	1.38	0.63–3.03	0
Sexual intercourse	3	3	1.50	0.97–2.32	45.3	3	3	1.43	0.91–2.23	0
Community										
Access to means	Data uncomplete					**1**	**1**	**1.6**	**1.04–2.45**	**NA**
Any social support	5	12	1	0.88–1.13	57.7	5	12	0.97	0.91–1.02	0
Family support	4	5	1.12	0.89–1.41	70.5	4	5	0.95	0.90–1.01	0
Peer support	3	3	1.1	0.88–1.38	21.3	3	3	1.17	0.80–1.70	45.3
Social support	3	4	0.76	0.56–1.04	38.0	3	4	1	0.72–1.39	15.3
Suicidal behavior of a friend	2	2	0.85	0.14–5.01	70.1	**2**	**2**	**1.65**	**1.07–2.56**	**0**
*Suicide death*										
Individual negative life events and family adversity										
Any negative life event^a^	**2**	**3**	**1.99**	**1.08–3.68**	**32.1**	**2**	**3**	**2.56**	**1.65–3.97**	**0**
Childhood maltreatment	**1**	**2**	**11.2**	**1.71–73.21**	**0**	**1**	**2**	**33.77**	**6.43–117.42**	**0**
Dysfunctional family	Data uncomplete					2	2	2.05	0.74–5.72	87.2
Family history of mental disorders and abuse	Data uncomplete					2	2	0.70	0.04–11.80	79.6
Family previous suicidal behavior	**1**	**2**	**5.68**	**1.51–21.38**	**4.9**	**1**	**2**	**7.03**	**2.79–17.71**	**0**
Psychiatric and psychological										
Psychiatric										
Antisocial disorder	No data					**1**	**6**	**4.19**	**2.31–7.61**	**19.9**
Any mental disorder or abuse	**2**	**8**	**3.64**	**1.11–11.88**	**50.9**	**2**	**11**	**4.92**	**3.52–6.87**	**0**
Conduct disorder	1	3	1.58	0.42–5.97	0	**2**	**3**	**5.02**	**1.91–13.15**	**0**
Drug abuse	Data uncomplete					**2**	**2**	**5.26**	**2.27–12.19**	**0**
Community										
Access to means	1	5	2.81	0.60–13.12	99.4	**2**	**4**	**4.00**	**3.69–4.34**	**0**

*95% CI* 95% confidence intervals, *OR* odds ratio, *PTSD* posttraumatic stress disorder, *ADHD* attention deficit hyperactivity disorder, *NSSI* non-suicidal self-injuries, *NA* not applicable

^a^Death of a parent, parental divorce, losing boy/girlfriend, trauma exposure, major events

#### Suicide attempts

##### Individual negative life events and family adversity

Risk factors for suicide attempts common to both genders included bullying (females: OR 6.30, 95% CI 1.53–25.90; males: OR 3.8, 95% CI 1.01–14.30), childhood maltreatment (females: OR 3.77, 95% CI 2.13–6.68; males: OR 2.76, 95% CI 1.20–6.36), community violence (females: OR 1.68, 95% CI 1.42–1.99; males: OR 1.83, 95% CI 1.48–2.26), and a family history of mental disorders, alcohol or drug abuse (females: OR 2.27, 95% CI 1.78–2.89; males: OR 2.63, 95% CI 1.99–3.47). Dating violence (OR 2.19, 95% CI 1.29–3.71) and having experienced interpersonal difficulties were associated with higher rates of suicide attempts in females (OR 1.13, 95% CI 1.03–1.24). Parental separation or divorce (OR 1.56, 95% CI 1.01–2.41) and previous suicidal behavior in the family (OR 2.84, 95% CI 1.87–4.33) were associated with suicide attempts only among males.

##### Psychiatric and psychological

The risk factors for suicide attempts, common to both genders, included previous suicidal ideation (females: OR 4.39, 95% CI 2.31–8.34; males: OR 3.97, 95% CI 1.40–11.24), previous suicide attempts (females: OR 6.96, 95% CI 3.75–12.91; males: OR 31.33, 95% CI 9.36–104.88), and a history of any mental disorder (females: OR 3.37, 95% CI 2.52–4.51; males: OR 4.27, 95% CI 3.28–5.47), specifically anxiety disorder (females: OR 2.03, 95% CI 1.77–2.33; males: OR 3.79, 95% CI 2.05–7.01), major depressive disorder (MDD) (females: OR 4.49, 95% CI 2.18–9.23; males: OR 6.07, 95% CI 1.74–21.20), and personality disorders (females: OR 7.89, 95% CI 3.81–16.35; males: OR 5.13, 95% CI 2.63–10.01). Other risk factors were alcohol abuse (females: OR 2.69, 95% CI 1.32–5.50; males: OR 2.14, 95% CI 1.09–4.20) and drug abuse (females: OR 4.44, 95% CI 2.51–7.83; males: OR 3.11, 95% CI 2.01–4.84).

Factors that increased the risk of suicide attempts only among females were bipolar disorder (OR 1.43, 95% CI 1.20–1.70), eating disorders (OR 5.27, 95% CI 2.04–13.60), posttraumatic stress disorder (PTSD) (OR 2.96, 95% CI 1.32–6.62), and depressive symptoms (OR 1.15, 95% CI 1.04–1.28). Factors significantly associated with suicide attempts among males were disruptiveness (OR 8.78, 95% CI 2.77–27.84) and hopelessness (OR 1.74, 95% CI 1.04–2.94).

##### Personal

Among females, a previous abortion significantly increased the risk of suicide attempts (OR 1.3, 95% CI 1.09–1.55).

##### Community

Male adolescents and young adults with access to means (e.g., firearms, pesticides, toxic gas) had a significant OR for suicide attempts compared with those who did not (OR 1.6, 95% CI 1.04–2.45). Exposure to the suicidal behavior of a friend (OR 1.65, 1.07–2.56) was significantly associated only in males.

#### Suicide death

##### Individual negative life events and family adversity

For both genders, any negative life event (e.g., death of a parent, losing boy/girlfriend) was a common risk factor for suicide death (females: OR 1.99, 95% CI 1.08–3.68; males: OR 2.56, 95% CI 1.65–3.97). Other factors were childhood maltreatment (females: OR 11.20, 95% CI 1.71–73.21; males: OR 33.77, 95% CI 6.43–177.22) and previous suicidal behavior in the family (females: OR 5.68, 95% CI 1.51–21.38; males: OR 7.03, 95% CI 2.79–17.71).

##### Psychiatric and psychological

Among both genders, the risk of suicide death was increased by a history of any mental disorder or abuse (females: OR 3.64, 95% CI 1.11–11.18; males: OR 4.92, 95% CI 3.52–6.87). Among males, significant associations were found with conduct disorder (OR 5.02, 95% CI 1.91–13.15), antisocial disorder (OR 4.19, 95% CI 2.31–7.61), and drug abuse (OR 5.26, 95% CI 5.26; 2.27–12.19).

##### Community

Among males, the risk of suicide was increased by access to means (OR 4.00, 95% CI 3.69–4.34). Among females, the risk was also increased, but not significantly so.

For both genders, nonsignificant associations were observed between the following risk and protective factors for suicide attempts: any negative life event, conflicts with the partner, attention deficit hyperactivity disorder, alcohol and drug use, conduct disorder, gambling disorder, non-suicidal self-injuries, low self-esteem, any kind of support, and all the personal factors assessed except abortion. For suicide death, nonsignificant associations were found with having a dysfunctional family and a family history of mental disorders.

## Discussion

We estimated the pooled risk of suicidal behaviors among adolescents and young adults and found that females had an almost twofold higher risk of suicide attempts than males, while males had an almost threefold higher risk of dying by suicide than females. Our meta-analysis has identified risk factors for both suicide attempts and death, which are common to male and female adolescents and young adults: exposure to any form of interpersonal violence and a history of mental or substance abuse disorder. Risk factors for suicide attempts included a history of previous suicidal thoughts and behaviors and a family history of mental disorders and abuse. For suicide death, a common risk factor was a family history of suicidal behavior. We also identified risk factors for suicide attempts in adolescents and young adults that were more specific for females or males, and for suicide death, which were specific for males only (Table 3). Finally, no significant associations were found between the protective factors assessed and suicide attempts and death.

**Table 3:** Significant meta-analyses results of gender risk factors of suicidal behavior among adolescents and young adults—results of the systematic review of gender differences in suicidal behavior in adolescents and young adults (covered up until January 2017)

Factor(s)	Severity^a^			
	Suicide death		Suicide attempt	
	Female	Male	Female	Male
*Individual negative life events and family adversity*				
Childhood maltreatment	**+++**	**+++**	++	++
Family previous suicidal behavior	**+++**	**+++**		++
Any negative life event^b^	+	++		
Bullying			**+++**	**++**
Family history of mental disorders and abuse			++	++
Community violence			+	+
Parental separation or divorce				+
*Psychiatric and psychological*				
Any mental disorder or abuse	++	++		
Drug abuse		**+++**		
Conduct disorder		**++**		
Antisocial disorder		++		
Major depressive disorder			++	+++
Personality disorder			+++	+++
Previous suicide attempts			+++	+++
Anxiety disorder			++	++
Alcohol abuse			++	++
Drug abuse			++	++
Previous suicidal ideation			++	++
Eating disorder			+++	
PTSD			++	
Dating violence			++	
Bipolar disorder			+	
Interpersonal difficulties			+	
Depressive symptoms			+	
Disruptiveness				**+++**
Hopelessness				+
*Personal*				
Abortion			+	
*Community*				
Suicidal behavior of a friend				+
Access to means		++		+

*PTSD* posttraumatic stress disorder

^a^Severity according to odds ratio values + > 1 to < 2, ++ ≥ 2 to < 5, +++ ≥ 5. ^b^Death of a parent, parental divorce, losing boy/girlfriend, trauma exposure, major events

### Gender as a risk factor for suicidal behaviors

Girls aged between 12 and 24 years have a higher lifetime prevalence (Evans et al. 2005; Kokkevi et al. 2012; Nock et al. 2013) and 12-month incidence (Evans et al. 2005; Afifi et al. 2007) of suicide attempts. The incidence and lethality of suicide attempts might be reduced among female youths by identifying high risk cases. Young women may be more likely to engage in help-seeking behaviors, to have a general readiness to talk about emotional problems (Beautrais 2002) and to frequently identify friends and professionals as sources of help (Rickwood et al. 2005). Moreover, considering that there is a high prevalence of mental disorders among youth who die by suicide (Renaud et al. 2008), help-seeking behaviors and contact with the health care system may diminish the risk of suicide among girls (Rhodes 2013).

In line with previous studies (Canetto and Sakinofsky 1998; Beautrais 2002), our results show that male youths have a considerably higher risk than females of dying by suicide. Higher mortality among males might be explained by the use of more lethal means, such as firearms and hanging methods (Beautrais 2003; Rhodes et al. 2014b), while drug poisoning is more frequent in females (Beautrais 2003; Mergl et al. 2015). Young males may be less predisposed to help-seeking behaviors in an attempt to exhibit masculine behaviors (Rhodes et al. 2014a). This association may be moderated by intentionality, impulsiveness, and aggressiveness (Beautrais 2003). Furthermore, a male tendency to adopt avoidance strategies (Gould et al. 2004) might make it more difficult for them to cope with emotional and behavioral problems.

An additional explanation for gender differences in suicide deaths may be misclassification. Suicide deaths tend to be reported as accidental or underdetermined due to shame, stigma, or lack of evidence (Beautrais et al. 1996). However, in a Canadian study that reclassified accidental or underdetermined deaths and suspected suicides, the gender gap of suicide rates remained for youths aged 16–25 years (Gould et al. 2004).

### Common and gender-specific risk factors for suicidal behaviors

#### Common risk factors

For suicide attempts, risk factors common to both genders include bullying, childhood maltreatment, community violence, previous suicidal thoughts and behaviors, any previous mental disorder or alcohol or drug abuse, and a family history of mental disorders and substance abuse. For suicide death, common risk factors include childhood maltreatment, any negative life events, and a family history of suicidal behavior.

Early exposure to traumatic life events, such as childhood maltreatment and bullying, implies complex processes that may increase vulnerability for suicidal behaviors, in both genders (Wilcox et al. 2009), including psychopathology (e.g., PTSD) (Wilcox et al. 2010) or maladaptive personality features (O´Brien and Sher 2013). Specifically, exposure to any childhood physical and/or psychological abuse is associated with a lack of social support and risky health behaviors, which consequently are related to poorer mental health and well-being (Sheikh et al. 2016). However, it seems that childhood traumatic experiences favor the development of internalizing symptoms in adulthood due to dissatisfaction with social connections more than a real lack of external support (Sheikh 2018). Furthermore, our findings agree with the results of an extended study conducted in eight eastern European countries, showing that individuals with traumatic childhood experiences were at a significantly increased risk of health-harming behaviors including suicide attempts (Bellis et al. 2014). We found an association between PTSD and suicide attempts among females, and the single study with males showed a threefold risk, which was statistically nonsignificant, probably due to the scarcity of data. No data were found to estimate the association between PTSD and suicide death.

A history of previous suicidal thoughts and behaviors is one of the most frequent common risk factors for later suicide attempts (Borges et al. 2008; O’Connor et al. 2015) and death (Suokas et al. 2001; Wenzel et al. 2011), as well as the presence of any mental disorder (Cavanagh et al. 2003; Zubrick et al. 2016), and alcohol and drug abuse (Evans et al. 2004) for both genders. Suicidal ideation has been related to MDD; when this relationship was analyzed, the risk of suicide attempts was higher among female adolescents and young adults (Wittchen 1994), especially among younger girls (Bolger et al. 1989). This association may also be moderated by depressive symptoms. In males, a predisposition to suicidal behavior may be moderated by hopelessness traits, disruptiveness and conduct problems, and antisocial disorders (highly related to aggressiveness).

Finally, strong associations were found between suicidal behavior in youths and exposure to a history of mental disorders or substance abuse or previous suicidal behaviors in family members. Vulnerability in youths with a family history of mental disorders or suicidal behavior may be reflected in their tendencies to experience increased rates of mental or substance abuse disorders and suicidal behaviors (Mann et al. 1999).

#### Female-specific risk factors

Female adolescent and young adult victims of dating violence are at a higher risk of attempting suicide. This risk might be moderated by a higher predisposition to have internalizing symptoms and a higher exposure to psychological abuse (Temple et al. 2016). Dating violence might also be a mediator in the association between abortion and suicidal thoughts in youths, the magnitude of this association being related to the severity of the aggression (Ely et al. 2009), but there is no evidence of any mechanism. Nevertheless, there are no similar data in relation to suicidal behaviors.

Previous studies, including a systematic review, are in agreement with our meta-analysis results showing previous abortion as risk factor for suicide attempts. This association may be moderated by mental disorders or substance use (Mota et al. 2010; Coleman 2011). Mental disorders could be related to poor social support or psychological factors that lead to unintended pregnancy; due to a feeling of inability to cope with pregnancy, women decide to have an abortion (Mota et al. 2010). Another possibility is that some vulnerability factors (e.g., poor social support) related to abortion and mental disorders mediate the association (Fergusson et al. 2006). Finally, interpersonal difficulties are associated with suicide attempts among female youths. This may be explained by their greater predisposition to emotional problems, increasing the risk (Kaess et al. 2011). It is clear that all the factors discussed are both interrelated and related to the occurrence of suicidal behaviors. Further research is needed to clarify the pathways and mechanisms.

#### Male-specific risk factors

According to our results, access to means was a relevant risk factor among male adolescents and young adults, for both suicide attempts and death. Male-specific risk factors for suicide attempts included parental separation or divorce. Our findings are consistent with evidence that living in single-parent families may increase the risk of suicide attempts in male youths. However, other reports suggest that females are also at risk (Chau et al. 2014; Dieserud et al. 2015) or that the risks are similar in both genders (Fergusson and Lynskey 1995; Kim and Kim 2008). In addition, disruptiveness, hopelessness, and previous suicidal behavior among family or friends increased the risk of suicide attempts among males. For suicide death, externalizing disorders and drug abuse conferred a significant risk.

Previous research has shown that male adolescents tend to have slightly more symptoms of externalizing problems, such as aggressive, delinquent (Kaess et al. 2011), and antisocial behavior (Marmorstein and Iacono 2005), which may act as mediators for suicidal behaviors. Further research is needed on this topic. In addition, similar to our data, some studies have found higher rates of suicide attempts among individuals exposed to suicidal behavior in the family and peers (Randall et al. 2015), showing the influence of the environment in youths.

#### Protective factors

No evidence on protective factors for suicidal behaviors was found in either males or females, probably due to the scarcity of published data. A previous study has shown that the risk of suicidal behavior in adolescents of both genders is reduced by family support (Tseng and Yang 2015) and is possibly increased by weak relationships with peers. In general, females have a higher perception of peer support than males (Kerr et al. 2006). Our meta-analyses results did not find a protective association between peer support and suicidal behaviors in both genders. However, the primary data used for the analyses reported peer support but not perception of it. In addition, peer support might not always be positive, since close relationships with peers involved in suicidal behaviors or at high risk of it do not act as a protective factor (Prinstein et al. 2010). Further investigation is needed for the assessment of protective factors and suicidal behaviors in young people.

### Limitations

This review has some limitations. We used the most widely recommended databases for psychiatric research, including Web of Science and PsycINFO (Löhönen et al. 2010), but were not able to search all available databases. Similar to previous systematic reviews (Devries et al. 2013; Maxwell et al. 2015), articles included came from a broad search strategy. Important information about vulnerable populations (e.g., incarcerated, veteran or active duty populations) was not considered because the inclusion criteria excluded institutionalized populations. No assessment was made of the suicide risk related to sexual orientation and gender identity. However, data analyzing these issues were already published (Miranda-Mendizábal et al. 2017).

The NOS was used to assess the quality of the included studies, but there is limited evidence on its validity (Wells et al. 2013). Nevertheless, its use is recommended by the Cochrane Collaboration. Random effect models were used for meta-analyses. They provide a very conservative estimate of the combined data with wider confidence intervals, as may be seen in some of our results. In addition, they may also lead to statistical values that are less likely to be significant (Borenstein et al. 2009).

For the association of gender and suicide death, only one cohort study was found, including individuals discharged from emergency departments; however, reference individuals were randomly selected from the general population, fulfilling our inclusion criteria. The wide heterogeneity observed in the meta-analyses of risk and protective factors may be explained by (1) the inclusion of observational studies that may have design flaws or tend to distort the magnitude or direction of associations (Stroup et al. 2000); (2) the differences in the adjustment; and (3) the possible reporting bias of the included studies. In addition, there were not enough studies to conduct meta-analyses for some risk, and especially, protective factors, particularly for suicide death.

### Implications for prevention

From a public health perspective, there is a need for the development and implementation of effective health policies and preventive strategies for suicidal behavior in adolescents and young adults, as well as for the early identification and reduction in the most prevalent risk factors. For example, reducing the different forms of interpersonal violence could help to diminish the prevalence of mental disorders and risky health and sexual behaviors (Wasserman et al. 2010). In addition, encouraging healthy behaviors (e.g., physical activity) may protect against some risk factors for suicide (Sheikh 2018). However, there is evidence that targeting individuals to change their behaviors will fail as long as the primary risk factors (e.g., childhood maltreatment) remain, because they would allow the appearance of new mediators (Sheikh et al. 2016).

Individual perception of social isolation may lead to impaired mental health and well-being. Strategies applying a more comprehensive approach (including community, school and family environment) (Fountoulakis et al. 2011) and increasing knowledge, to facilitate help-seeking behaviors, could be more effective (Riner and Saywell 2002). In addition, rather than implementing gender-specific prevention strategies, it is important for strategies to target and better address the most prevalent risk and protective factors to prevent suicidal behaviors.

### Implications for research

Although gender differences in youth suicidal behavior have been identified, further research is needed. We encourage longitudinal research assessing the role of sociodemographic variables (e.g., socioeconomic status, ethnicity) in suicidal behavior among young persons. Additional research is also needed on academic (e.g., academic failure) and protective factors (e.g., resilience) in young females and males, as well as research on access to means, externalizing problems, and a family history of mental disorders and abuse among young females, and relationship problems, bipolar and eating disorders in young males. To reduce suicide mortality, information is needed on related pathways in both genders. Importantly, the development and implementation of preventive strategies should include gender preferences and context. To do so, youth preferences with respect to public health interventions should be assessed. Finally, as gender is one of the most important social determinants of health inequalities (Solar and Irwin 2010), efforts should be made to reduce the gender gap in health issues, particularly during adolescence and young adulthood, which are periods of special vulnerability.

## Electronic supplementary material

Below is the link to the electronic supplementary material.

Supplementary material 1 (DOCX 1292 kb)
