# Supplementary material for: Gender differences in suicidal behavior in adolescents and young adults: systematic review and meta-analysis of longitudinal studies
Source: Int J Public Health. 2019 Jan 12;64(2):265–83. doi: 10.1007/s00038-018-1196-1 (PMC6439147; doi:10.1007/s00038-018-1196-1)
Supplement: Supplementary file 1 — Supplementary material 1 (DOCX 1292 kb) [file 38_2018_1196_MOESM1_ESM.docx]

**Journal:** International Journal of Public Health

**Gender Differences in Suicidal Behavior in Adolescents AND YOUNG ADULTS: Systematic Review and Meta-Analysis of Longitudinal Studies**

**TABLE S1.** MOOSE checklist of the systematic review of gender differences in suicidal behavior in adolescents and young adults (covered up until January 2017)

| **Reporting of background should include** |  |
| --- | --- |
| Problem definition | **√** |
| Hypothesis statement |  |
| Description of study outcome(s) | **√** |
| Type of exposure or intervention used | **√** |
| Type of study designs used | **√** |
| Study population | **√** |
| **Reporting of search strategy should include** |  |
| Qualifications of searchers (eg. librarians and investigators) | **√** |
| Search strategy, including time period included in the synthesis and keywords | **√** |
| Effort to include all available studies, including contact with authors | **√** |
| Databases and registries searched | **√** |
| Search software used, name and version, including special features used (eg, explosion) | **√** |
| Use of hand searching (eg, reference lists of obtained articles) | **√** |
| List of citations located and those excluded, including justification | **√** |
| Method of addressing articles published in languages other than English | **√** |
| Method of handling abstracts and unpublished studies | **√** |
| Description of any contact with authors | **√** |
| **Reporting of methods should include** |  |
| Description of relevance of appropriateness of studies assembled for assessing the hypothesis to be tested | **√** |
| Rationale for the selection and coding data (eg, sound clinical principles or convenience) | **√** |
| Documentation of how data were classified and coded (eg, multiple raters, blinding, and interrater reliability) | **√** |
| Assessment of confounding (eg, comparability of cases and controls in studies where appropriate) | **√** |
| Assessment of study quality, including blinding of quality assessors; stratification or regression on possible predictors of study results | **√** |
| Assessment of heterogeneity | **√** |
| Description of statistical methods (eg, complete description of fixed or random effects models, justification of whether the chosen models account for predictors of study results, dose-response models, or cumulative meta-analysis) in sufficient detail to be replicated | **√** |
| Provision of appropriate tables and graphics | **√** |
| **Reporting of results should include** |  |
| Graphic summarizing individual study estimates and overall estimate | **√** |
| Table giving descriptive information for each study included | **√** |
| Results of sensitivity testing (eg, subgroup analysis) | **√** |
| Indication of statistical uncertainty of findings | **√** |
| **Reporting of discussion should include** |  |
| Quantitative assessment of bias (eg, publication bias) | **√** |
| Justification for non-exclusion (eg, exclusion of non-English-language citations) | **√** |
| Assessment of quality of included studies | **√** |
| **Reporting of conclusion should include** |  |
| Consideration of alternative explanations for observed results | **√** |
| Generalization of the conclusion (i.e., appropriate for the data presented and within the domain of the literature review) | **√** |
| Guidelines for future research | **√** |
| Disclosure of funding source | **√** |

| **TABLE S2.** Quality of assessment of included articles in the systematic review of gender differences in suicidal behavior in adolescents and young adults (covered up until January 2017)* | | | | | | | | | |
| --- | --- | --- | --- | --- | --- | --- | --- | --- | --- |
|  | | | | | | | | | |
| Study | Domains | | | | | | | |  |
|  | Selection | | | | Comparability | Outcome | | |  |
|  | Representativeness of Exposed Cohort^¥^ | Selection of Non Exposed Cohort^¥^ | Ascertainment of Exposure^¥^ | Demonstration Outcome of Interest Not Present at Start of Study^¥^ | Comparability of Cohorts on the basis of Design or Analysis^ǂ^ | Ascertainment of Outcome^¥^ | Adequate Length of Follow Up^¥^ | Adequacy of Follow Up^¥^ | Total stars |
| **Cohort studies** |  |  |  |  |  |  |  |  |  |
| Kaplan H & Pokonny A, 1976 | ***** | ***** | _ | ***** | ***** | _ | _ | _ | 4 |
| Reinherz HZ et al. 1995 | * | * | * | * | ** | _ | * | * | 8 |
| Silverman AB et al. 1996 | * | * | * | * | * | * | * | * | 8 |
| McKeown RE et al. 1998 | * | * | * | _ | * | _ | _ | _ | 4 |
| Wichstrom L. 2000 | * | * | _ | _ | * | _ | _ | * | 4 |
| Borowsky I et al. 2001 | * | * | * | * | ** | _ | _ | _ | 5 |
| Lewinsohn P et al. 2001 | * | * | * | * | ** | * | * | _ | 8 |
| Sourander A et al. 2001, 2009 | * | * | * | _ | * | _ | * | _ | 5 |
| Fergusson D et al. 2003 | * | * | * | * | ** | _ | * | * | 8 |
| Bearman P et al. 2004 | * | * | * | * | ** | ₋ | ₋ | * | 7 |
| Ialongo NS et al. 2004 | * | * | * | * | ** | * | * | _ | 8 |
| D´Augelli A et al. 2005 | * | * | * | * | * | _ | _ | _ | 5 |
| Feigelman W et al. 2006, Thompson M et al. 2007; Exner-Cortens D et al. 2013; Van Dulmen M et al. 2013; Abrutyn S et al. 2014; Turanovic JT & Pratt TC 2015 | * | * | * | * | ** | _ | * | * | 8 |
| Kidd S et al. 2006 | * | * | _ | _ | * | _ | _ | _ | 3 |
| Rodríguez-CanoT et al. 2006 | _ | * | * | * | * | _ | * | _ | 5 |
| Ackard D et al. 2007 | * | * | * | * | ** | _ | _ | * | 7 |
| Brezo J et al. 2007, 2008 | * | * | * | * | ** | _ | * | _ | 7 |
| Crow S et al. 2008 | * | * | _ | * | ** | _ | * | * | 7 |
| Dupéré V et al. 2008 | * | * | * | _ | * | _ | * | _ | 5 |
| Lambert SF et al., 2008 | _ | * | _ | * | ** | _ | * | * | 6 |
| Nrugham L et al. 2008, 2015 | * | * | * | * | ** | _ | * | _ | 7 |
| Wong J et al. 2008 | * | * | * | * | * | _ | _ | _ | 5 |
| Study | Domains | | | | | | | |  |
|  | Selection | | | | Comparability | Outcome | | | |
|  | Representativeness of Exposed Cohort^¥^ | Selection of Non Exposed Cohort^¥^ | Ascertainment of Exposure^¥^ | Demonstration Outcome of Interest Not Present at Start of Study^¥^ | Comparability of Cohorts on the basis of Design or Analysis^ǂ^ | Ascertainment of Outcome^¥^ | Adequate Length of Follow Up^¥^ | Adequacy of Follow Up^¥^ | Total stars |
| Wilcox H et al. 2009 | * | * | * | * | * | _ | * | _ | 6 |
| Batty G et al. 2010 | * | * | * | * | ** | * | * | * | 9 |
| Peter T et al. 2010 | * | * | * | * | * | _ | _ | _ | 5 |
| Roberts RE et al. 2010 | * | * | * | * | ** | _ | _ | * | 7 |
| Klomek A et al. 2011 | * | * | * | * | ** | * | * | * | 9 |
| Young R et al. 2011 | * | * | * | * | ** | _ | * | * | 8 |
| Fried L et al. 2012 | * | * | * | * | * | _ | _ | _ | 5 |
| Guan K et al. 2012 | * | * | * | * | * | _ | _ | _ | 5 |
| Hurtig T et al. 2012 | * | * | _ | * | * | _ | * | _ | 5 |
| Nkansah-Amankra S et al. 2012 | * | * | * | * | ** | _ | * | _ | 7 |
| Wanner B et al. 2012 | * | * | * | * | ** | _ | * | _ | 7 |
| Winterrowd E & Canetto SS, 2013 | * | * | * | * | ** | _ | _ | * | 7 |
| Chang S-S et al. 2014 | * | * | * | * | ** | _ | * | _ | 7 |
| Chuan-Yu C et al. 2014 | * | _ | * | * | ** | * | * | * | 8 |
| Conner K et al. 2014 | * | * | * | * | * | _ | _ | * | 6 |
| Luntano T et al. 2014 | * | * | * | * | ** | * | * | * | 9 |
| Mars B et al. 2014 | * | * | * | * | * | _ | * | _ | 6 |
| Miranda R et al. 2014 | * | * | * | * | ** | _ | * | _ | 7 |
| Soller B et al. 2014 | * | * | * | * | ** | _ | * | _ | 7 |
| Swanson E et al. 2014 | * | * | _ | * | ** | _ | * | * | 7 |
| Finkelstein Y et al. 2015 | * | * | * | * | * | * | * | * | 8 |
| Scott L et al. 2015 | * | * | * | * | ** | _ | * | * | 8 |
| You J et al. 2015 | * | * | * | * | ** | _ | _ | * | 7 |
| Conway PM et al. 2016 | * | * | * | * | * | * | _ | * | 7 |
| Feigelman W et al. 2016 | * | * | * | * | * | * | * | _ | 7 |
| Meza JI et al. 2016 | * | * | * | _ | ** | _ | * | * | 7 |
| Mok P et al. 2016 | * | * | * | * | * | * | * | * | 8 |
| Weiser et al. 2016 | * | * | * | * | ** | _ | * | * | 8 |
| Hishinuma ES et al. 2017 | * | * | _ | * | * | _ | * | * | 6 |
| Study | Case Definition^¥^ | Representativeness of Cases^¥^ | Selection of Controls^¥^ | Definition of Controls^¥^ | Comparability of Cases and Controls^ǂ^ | Ascertainment of Exposure^¥^ | Same Method Ascertainment Both Groups^¥^ | Non Response Rate^¥^ | Total of stars |
| **Case-control studies** |  |  |  |  |  |  |  |  |  |
| Salk L et al. 1985 | ***** | ***** | ***** | **_** | ***** | **_** | ***** | **_** | 5 |
| King CA et al. 1990 | **_** | ***** | ***** | ***** | ***** | **_** | ***** | **_** | 5 |
| Rotheram-Borus M & Shrout P, 1990 | ***** | ***** | ***** | ***** | ***** | **_** | **_** | **_** | 5 |
| Garnefski N et al. 1992 | _ | * | * | * | ** | _ | * | * | 7 |
| Brent DA et al. 1993, 1999 | _ | * | * | * | ** | _ | * | _ | 6 |
| Adams D et al. 1994 | _ | * | * | _ | ** | * | * | _ | 6 |
| Shaffer D et al. 1996 | * | * | _ | * | ** | _ | * | * | 7 |
| Beautrais A et al. 1998 | * | * | * | * | ** | _ | * | _ | 7 |
| Lyon ME et al. 2000 | _ | _ | _ | * | ** | _ | * | _ | 4 |
| Ikeda RM et al. 2001 | * | * | * | * | ** | _ | * | _ | 7 |
| Donald M et al. 2005 | _ | * | * | * | ** | _ | _ | _ | 5 |
| Ostry A et al. 2007 | * | * | * | * | ** | * | * | * | 9 |
| Bilgin M et al. 2007 | _ | _ | * | * | _ | _ | * | _ | 3 |
| Freitas G et al. 2008 | _ | * | * | * | * | _ | * | * | 6 |
| Christiansen E et al.2011, 2012 | * | * | * | * | ** | * | * | * | 9 |
| Cheng C-CJ et al. 2014 | * | * | * | * | * | * | * | * | 8 |
| *Highest quality studies are awarded up to nine stars. ^¥^A maximum of one star can be allotted in this category. ^ǂ^A maximum of two stars can be allotted in this category. _None star was allotted. | | | | | | | | | |

**SupplementaRy TEXT S1 of THE systematic review of gender differences in suicidal behavior in adolescents and young adults** (covered up until January 2017)

**Search strategy and selection criteria of the broader systematic review**

A broad-scope and inclusive initial search strategy was carried out, with no restrictions of population or age, in order to identify predictors of suicidal-related behaviors. Text-word, titles and Mesh terms were used as search terms resulting initially in 26,882 references after removal of duplicates (Fig. 1). Based on this search strategy other analyses in different populations and risk factors, are currently being carried out.

All the keywords used for inclusion and exclusion, as well as search terms used to identify suicide attempt, suicidal behavior, population and study design are provided (see below). The following databases were searched: Cochrane Library, Medline, PsychINFO, EMBASE and Web of Science. A search in grey literature was conducted using the OpenGrey database, and reference lists from previous reviews and books were examined. Searching in all databases covered up until January 2017. No restrictions of language or year of publication were applied. Corresponding authors for articles written in languages other than English and Spanish were contacted.

A multidisciplinary team of psychiatrists, psychologists, statisticians, epidemiologists and public health professionals was established to perform the review. Five groups of independent peer reviewers assessed all references. During title review discrepancies between reviewers were included. During title and abstract review phases, reviewers were blinded from seeing the article's author, journal and year of publication to minimize selection bias. A third independent reviewer resolved any discrepancies during abstract and full text review.

For the broad-scope review, studies were included if they met all of the following criteria: (a) reporting suicide death or attempt as dependent variable; (b) assessing at least one risk or protective factor of any of these outcomes; (c) study population age range between 12 and 26 years old, both inclusive; (d) population-based longitudinal studies (non-clinical and non-institutionalized sample cohorts; or case-control where control group was of the same age range and population-based). Ecological and cross-sectional studies were excluded. Using an expert consensus reported previously, suicide death (or simply, suicide) was defined as any fatal act done with the intention of taking one’s own life, while suicide attempt was defined as any act of self-injury with intention to die ^15^. Other suicide-related behaviors (e.g., suicide ideation) were excluded.

Using the listed criteria, 197 studies were identified for qualitative synthesis.

**Detailed search strategy**

**Search terms by database**

| **Components** | **Keywords** |  |
| --- | --- | --- |
|  | **Inclusion keywords** | **Exclusion keywords** |
| Population | Humans | Animals |
| Outcome | Suicide  Suicidal behavior  Suicide ideation  Suicide plan  Suicide attempt  Non-suicidal self-injury  Parasuicide  Self-injure  Deliberate self-harm  Suicidality  Non-fatal suicidal behavior |  |
| Exposure | Risk factor  Causality  Relationship  Association  Prediction  Harm  Adverse  Antecedent  History  Etiology  Protective factor  Prevention  Improvement  Prevalence  Incidence |  |
| Study design | Experimental study  Randomized controlled trial  Controlled clinical trial  Clinical trial  Longitudinal study  Observational study  Cohort study  Case control study  Time series study  Prospective study  Retrospective study  Follow-up  Cross-sectional study | Case series  Case report |
| Others |  | Type of publications:  - Comments  - Letter  - Editorial |

**Search Strategy in each selected database:**

1. **Medline (Pubmed): Searched January 17th, 2017**

|  | **Search Strategy** |
| --- | --- |
| #24 | (#20 AND #21 AND #22) NOT (#18 OR #19 OR #20) |
| #23 | (#14 OR #15 OR #16 OR #17) |
| #22 | (#10 OR #11 OR #12 OR #13) |
| #21 | (#5 OR #6 OR #7 OR #8 OR #9) |
| #20 | (#3 OR #4) |
| #19 | (#1 NOT (#1 AND #2)) |
| #18 | case reports[Publication Type] |
| #17 | (("prospective studies"[MeSH Terms] OR ("prospective"[All Fields] AND "studies"[All Fields]) OR "prospective studies"[All Fields] OR ("prospective"[All Fields] AND "study"[All Fields]) OR "prospective study"[All Fields]) OR ("retrospective studies"[MeSH Terms] OR ("retrospective"[All Fields] AND "studies"[All Fields]) OR "retrospective studies"[All Fields] OR ("retrospective"[All Fields] AND "study"[All Fields]) OR "retrospective study"[All Fields]) OR Follow-up[All Fields] OR ("cross-sectional studies"[MeSH Terms] OR ("cross-sectional"[All Fields] AND "studies"[All Fields]) OR "cross-sectional studies"[All Fields] OR ("cross"[All Fields] AND "sectional"[All Fields] AND "study"[All Fields]) OR "cross sectional study"[All Fields])) |
| #16 | (("cohort studies"[MeSH Terms] OR ("cohort"[All Fields] AND "studies"[All Fields]) OR "cohort studies"[All Fields] OR ("cohort"[All Fields] AND "study"[All Fields]) OR "cohort study"[All Fields]) OR ("case-control studies"[MeSH Terms] OR ("case-control"[All Fields] AND "studies"[All Fields]) OR "case-control studies"[All Fields] OR ("case"[All Fields] AND "control"[All Fields] AND "study"[All Fields]) OR "case control study"[All Fields]) OR (("time"[MeSH Terms] OR "time"[All Fields]) AND series[All Fields] AND ("clinical trials as topic"[MeSH Terms] OR ("clinical"[All Fields] AND "trials"[All Fields] AND "topic"[All Fields]) OR "clinical trials as topic"[All Fields] OR "study"[All Fields] OR "biomedical research"[MeSH Terms] OR ("biomedical"[All Fields] AND "research"[All Fields]) OR "biomedical research"[All Fields]))) |
| #15 | (("controlled clinical trial"[Publication Type] OR "controlled clinical trials as topic"[MeSH Terms] OR "controlled clinical trial"[All Fields]) OR ("clinical trial"[Publication Type] OR "clinical trials as topic"[MeSH Terms] OR "clinical trial"[All Fields]) OR ("longitudinal studies"[MeSH Terms] OR ("longitudinal"[All Fields] AND "studies"[All Fields]) OR "longitudinal studies"[All Fields] OR ("longitudinal"[All Fields] AND "study"[All Fields]) OR "longitudinal study"[All Fields]) OR (Observational[All Fields] AND ("clinical trials as topic"[MeSH Terms] OR ("clinical"[All Fields] AND "trials"[All Fields] AND "topic"[All Fields]) OR "clinical trials as topic"[All Fields] OR "study"[All Fields] OR "biomedical research"[MeSH Terms] OR ("biomedical"[All Fields] AND "research"[All Fields]) OR "biomedical research"[All Fields]))) |
| #14 | ((Experimental[All Fields] AND ("clinical trials as topic"[MeSH Terms] OR ("clinical"[All Fields] AND "trials"[All Fields] AND "topic"[All Fields]) OR "clinical trials as topic"[All Fields] OR "study"[All Fields] OR "biomedical research"[MeSH Terms] OR ("biomedical"[All Fields] AND "research"[All Fields]) OR "biomedical research"[All Fields])) OR ("randomized controlled trial"[Publication Type] OR "randomized controlled trials as topic"[MeSH Terms] OR "randomized controlled trial"[All Fields] OR "randomised controlled trial"[All Fields])) |
| #13 | (("risk factors"[MeSH Terms] OR ("risk"[Tiab] AND "factors"[Tiab]) OR "risk factors"[Tiab] OR ("risk"[Tiab] AND "factor"[Tiab]) OR "risk factor"[Tiab])) |
| #12 | (("etiology"[Subheading] OR "etiology"[Tiab] OR "causality"[Tiab] OR "causality"[MeSH Terms]) OR Relationship[Tiab] OR ("association"[MeSH Terms] OR "association"[Tiab]) OR Prediction[Tiab] OR Harm[Tiab] OR Adverse[Tiab] OR Antecedent[Tiab] OR ("history"[Subheading] OR "history"[Tiab] OR "history"[MeSH Terms])) |
| #11 | ((“protective factors”[Tiab] OR (“protective”[Tiab] AND “factor”[Tiab]) OR (“protective”[Tiab] AND “factors”[Tiab]) OR "prevention"[Tiab]) OR Improvement[Tiab]) |
| #10 | (prevalence[MeSH Terms] OR incidence[MeSH Terms] OR prevalence[Tiab] OR incidence[Tiab]) |
| #9 | (("deliberate"[All Fields] AND "self"[All Fields] AND "harm"[All Fields]) OR "deliberate self-harm"[All Fields]) |
| #8 | ((Non-suicidal[All Fields] AND self-injury[All Fields]) OR ("self-injurious behavior"[MeSH Terms] OR ("self-injurious"[All Fields] AND "behavior"[All Fields]) OR "self-injurious behavior"[All Fields] OR "parasuicide"[All Fields]) OR Self-injure[All Fields] OR ("self-injurious behavior"[MeSH Terms] OR ("self-injurious"[All Fields] AND "behavior"[All Fields]) OR "self-injurious behavior"[All Fields])) |
| #7 | ("suicide, attempted"[MeSH Terms] OR ("suicide"[All Fields] AND "attempted"[All Fields]) OR "attempted suicide"[All Fields] OR ("suicide"[All Fields] AND "attempt"[All Fields]) OR "suicide attempt"[All Fields]) |
| #6 | ((suicidal[All Fields] AND ("behaviour"[All Fields] OR "behavior"[MeSH Terms] OR "behavior"[All Fields])) OR (("suicide"[MeSH Terms] OR "suicide"[All Fields]) AND (ideation[All Fields] OR plan[All Fields]))) |
| #5 | (suicid*) OR "suicide"[MeSH Terms] OR "suicide"[All Fields]) |
| #4 | editorial [Publication Type] |
| #3 | letter[Publication Type] |
| #2 | human[MeSH Terms] |
| #1 | animal[MeSH Terms] |

1. **Embase: Searched January 17th, 2017**

|  | **Search Strategy** |
| --- | --- |
| #6 | (#3 AND #4 AND #5) NOT (#1 OR #2) |
| #5 | 'experimental study'/exp OR 'experimental study' OR 'randomized controlled trial'/exp OR 'randomized controlled trial' OR 'controlled clinical trial'/exp OR 'controlled clinical trial' OR 'clinical trial'/exp OR 'clinical trial' OR 'longitudinal study'/exp OR 'longitudinal study' OR 'observational study'/exp OR 'observational study' OR 'cohort analysis'/exp OR 'cohort analysis' OR 'case control study'/exp OR 'case control study' OR 'prospective study'/exp OR 'prospective study' OR 'retrospective study'/exp OR 'retrospective study' OR 'follow-up'/exp OR 'follow-up' OR 'cross-sectional study'/exp OR 'cross-sectional study' OR 'cohort study'/exp OR 'cohort study' OR 'time series study' |
| #4 | 'risk factor'/exp OR 'risk factor':ti,ab OR 'prediction'/exp OR 'prediction':ti,ab OR 'association'/exp OR 'association':ti,ab OR 'prevention'/exp OR 'prevention':ti,ab OR 'causality'/exp OR 'causality':ti,ab OR 'relationship':ti,ab OR 'adverse':ti,ab OR 'antecedent':ti,ab OR 'etiology'/exp OR 'etiology':ti,ab OR 'protective factor':ti,ab OR prevalence/exp OR incident/exp |
| #3 | suicid* OR 'suicidal behavior'/exp OR 'suicidal behavior' OR 'automutilation'/exp OR 'automutilation' OR 'suicide'/exp OR 'suicide' OR 'suicide ideation'/exp OR 'suicide ideation' OR 'suicide plan' OR 'suicide attempt'/exp OR 'suicide attempt' OR 'non-suicidal self-injury' OR 'parasuicide'/exp OR 'parasuicide' OR 'self-injure' OR 'deliberate self-harm' OR suicidality |
| #2 | letter/exp OR editorial/exp OR “case report”/exp |
| #1 | animal/exp NOT (animal/exp AND human/exp) |

1. **Web of Science: Search October 21, 2013**

|  | **Search Strategy** |
| --- | --- |
| #6 | (#3 AND #4 AND #5) NOT (#1 OR #2) |
| #5 | TS=(Experimental study OR Randomized controlled trial OR Controlled clinical trial OR Clinical trial OR Longitudinal study OR Observational study ORCohort study OR Case control study OR Time series study OR Prospective study OR Retrospective study OR Follow-up OR Cross-sectional study) |
| #4 | TS=(Risk factor OR Causality OR Relationship OR Association OR Prediction OR Harm OR Adverse OR Antecedent OR History OR $Etiology OR Protective factor OR Prevention OR Improvement OR prevalence OR incidence) |
| #3 | TS=(Suicid* OR suicide OR Suicidal behavior OR Suicide ideation OR Suicide plan OR Suicide attempt OR Non-suicidal self-injury OR Parasuicide OR Self-injure OR Deliberate self-harm OR Suicidality OR Non-fatal suicidal behaviour) |
| #2 | DT=(letter) OR TI=(editorial) OR TI=(case report) |
| #1 | TS=(animal NOT (animal AND human)) |

1. **The Cochrane Library: Search January 20th, 2017**

|  | **Search Strategy** |
| --- | --- |
| #39 | (#14 and #29 and #37) not (#3 or #38) |
| #38 | MeSH descriptor: [Case Reports] explode all trees |
| #37 | #30 or #31 or #32 or #33 or #34 or #35 or #36 |
| #36 | MeSH descriptor: [Cross-Sectional Studies] explode all trees |
| #35 | MeSH descriptor: [Case-Control Studies] explode all trees |
| #34 | MeSH descriptor: [Cohort Studies] explode all trees |
| #33 | MeSH descriptor: [Longitudinal Studies] explode all trees |
| #32 | MeSH descriptor: [Randomized Controlled Trials as Topic] explode all trees |
| #31 | MeSH descriptor: [Randomized Controlled Trial] explode all trees |
| #30 | MeSH descriptor: [Research Design] explode all trees |
| #29 | #15 or #16 or #17 or #18 or #19 or #20 or #21 or #22 or #23 or #24 or #25 or #26 or #27 or #28 |
| #28 | improvement:ti,ab,kw |
| #27 | prevention:ti,ab,kw |
| #26 | protective factor*:ti,ab,kw |
| #25 | etiology:ti,ab,kw |
| #24 | history:ti,ab,kw |
| #23 | antecedent:ti,ab,kw |
| #22 | adverse:ti,ab,kw |
| #21 | harm:ti,ab,kw |
| #20 | prediction:ti,ab,kw |
| #19 | association:ti,ab,kw |
| #18 | relationship:ti,ab,kw |
| #17 | MeSH descriptor: [Causality] explode all trees |
| #16 | risk factor*:ti,ab,kw |
| #15 | MeSH descriptor: [Risk] explode all trees |
| #14 | #2 or #3 or #4 or #5 or #6 or #7 #8 or #9 or #10 or #11 |
| #13 | Deliberat* self-harm |
| #12 | self-injur* |
| #11 | Parasuicide |
| #10 | Non-suicid* self-injury |
| #9 | suicid* attempt* |
| #8 | suicid* plan |
| #7 | suicid* ideation |
| #6 | suicid* behavi* |
| #5 | suicid* |
| #4 | MeSH descriptor: [Suicide] explode all trees |
| #3 | #2 not (#2 and #1) |
| #2 | MeSH descriptor: [Animals] explode all trees |
| #1 | MeSH descriptor: [Humans] explode all trees |

1. **PsycInfo (EBSCOHost): Search January 21th, 2017**

|  | **Search Strategy** |
| --- | --- |
| #1 | ((DE “suicidal ideation” OR Suicidal Ideation OR DE "Suicide+" OR DE "Assisted Suicide" OR (DE “suicidology”) OR suicid***** *OR* (suicidal AND behav*) OR “suicidal ideation” OR “suicide plan” *OR* “suicide attempt” OR “attempted suicide” *OR* suicidality *OR* **“**suicide prevention” *OR* parasuicide *OR* **“**self-injurious behavior” *OR* "non-suicidal self-injury” *OR* self-injur* *OR* "non-fatal suicidal behavior" *OR* "non-fatal suicidal behaviour" OR (DE "Suicide Prevention")) AND (DE "risk factors" OR “risk factors” OR DE “causality” OR (TI causalit* OR AB causalit*) OR (TI relationship OR AB relationship) OR (TI Association* OR AB Association*) OR DE "Prediction" OR DE "Harm Reduction" OR (TI adverse OR AB adverse) OR (TI History OR AB History) OR DE "Etiology" OR (DE "Protective Factors") OR (TI “Protective factors” OR AB “Protective factors”) OR (DE "Prevention") OR (DE "Accident Prevention") OR prevalence OR incidence OR (DE "Primary Mental Health Prevention") OR (DE "Relapse Prevention") OR (TI improve* OR AB improve*)) AND (DE "Clinical Trials+" OR DE "Experimental Design" OR DE "Between Groups Design" OR DE "Clinical Trials" OR DE "Cohort Analysis" OR DE "Followup Studies" OR DE "Hypothesis Testing" OR DE "Longitudinal Studies" OR DE "Repeated Measures" OR DE "Prospective Studies" OR DE “Case-control” OR DE “Cross-sectional” OR DE "Time Series" OR DE "Retrospective Studies")) NOT ((DE “Animals+” OR DE "Female Animals" OR DE "Infants (Animal)" OR DE “Invertebrates” OR DE "Male Animals" OR DE "Vertebrates") OR (DE “Case report”)) |

1. **OpenGrey: Searched January 20^th^, 2017**

|  | **Search Strategy** |
| --- | --- |
| #1 | ((suicide* OR (suicide* (behaviour OR behaviour)) OR (suicide* attempt*) OR (deliberate self-harm) OR (suicidality) OR self-injur*) AND (risk factor* OR causalit* OR relationship* OR association* OR prediction* OR harm* OR adverse OR antecedent* OR history OR etiology OR protective factor* OR prevention* OR improvement* OR incidence) AND (longitudinal study OR observational study OR cohort study OR ((case AND control) study) OR prospective study OR retrospective study OR “follow-up”) AND (young* OR youth OR child* OR adolescent* OR (college student*) OR (university student*) OR (young worker*)) |

**SupplementaRy TEXT S2 THE systematic review of gender differences in suicidal behavior in adolescents and young adults** (covered up until January 2017)

**References of included articles (n=77) (Studies n=67)**

1. Kaplan HB, Pokorny AD (1976) Self-Attitudes and Suicidal Behavior. Suicide Life-Threatening Behav 6:90–91
2. Reinherz HZ, Giaconia RM, Silverman AB, et al (1995) Early Psychosocial Risks for Adolescent Suicidal Ideation and Attempts. J Am Acad Child Adolesc Psychiatry 34:599–611. doi: 10.1097/00004583-199505000-00012
3. Silverman AB, Reinherz HZ, Giaconia RM (1996) The long-term sequelae of child and adolescent abuse: A longitudinal community study. Child Abus Negl 20:709–723. doi: 10.1016/0145-2134(96)00059-2
4. McKeown RE, Garrison CZ, Cuffe SP, et al (1998) Incidence and predictors of suicidal behaviors in a longitudinal sample of young adolescents. J Am Acad Child Adolesc Psychiatry 37:612–619
5. Wichstrøm L (2000) Predictors of adolescent suicide attempts: a nationally representative longitudinal study of Norwegian adolescents. J Am Acad Child Adolesc Psychiatry 39:603–610. doi: 10.1097/00004583-200005000-00014
6. Borowsky I, Ireland M, Resnick M (2001) Adolescent suicide attempts: risks and protectors. Pediatrics 107:485–493. doi: 10.1542/peds.107.3.485
7. Lewinsohn P (2001) Gender differences in suicide attempts from adolescence to young adulthood. J Am Acad Child Adolesc Psychiatry 40:427–434
8. Sourander A, Helstelä L, Haavisto A, Bergroth L (2001) Suicidal thoughts and attempts among adolescents: A longitudinal 8-year follow-up study. J Affect Disord 63:59–66. doi: 10.1016/S0165-0327(00)00158-0
9. Sourander A, Brunstein Klomek A, Niemelä S, et al (2009) Childhood Predictors of Completed and Severe Suicide Attempts. Arch Gen Psychiatry 66:398–406
10. Fergusson D, Beautrais A, Horwood L (2003) Vulnerability and resiliency to suicidal behaviours in young people. Psychol Med 33:61–73. doi: 10.1017/S0033291702006748
11. Bearman PS, Moody J (2004) Suicide and Friendships Among American Adolescents. Am J Public Health 94:89–95
12. Ialongo NS, Mcnaught ALK, Wagner BM, et al (2004) African American Children’s Reports of Depressed Mood , Hopelessness , and Suicidal Ideation and Later Suicide Attempts. Suicide Life-Threatening Behav 34:395–407
13. D´Augelli AR, Grossman AH, Salter NP, et al (2005) Predicting the Suicide Attempts of Lesbian, Gay, and Bisexual Youth. Suicide Life-threatening Behav 35:646–660
14. Feigelman W, Gorman BS, Lesieur H (2006) Examining the Relationship Between At-Risk Gambling and Suicidality in a National Representative Sample of Young Adults. Suicide Life-threatening Behav 36:396–409
15. Thompson MP, Ho C, Kingree JB (2007) Prospective Associations between Delinquency and Suicidal Behaviors in a Nationally Representative Sample. J Adolesc Heal 40:232–237. doi: 10.1016/j.jadohealth.2006.10.016
16. Exner-Cortens D, Eckenrode J, Rothman E (2013) Longitudinal associations between teen dating violence victimization and adverse health outcomes. Pediatrics 131:71–78. doi: 10.1542/peds.2012-1029
17. Van Dulmen M, Mata A, Claxton S, et al (2013) Longitudinal associations between violence and suicidality from adolescence into adulthood. Suicide Life-Threatening Behav 43:523–531. doi: 10.1111/sltb.12036
18. Abrutyn S, Mueller AS (2015) Are Suicidal Behaviors Contagious in Adolescence?: Using Longitudinal Data to Examine Suicide Suggestion*. Am Sociol Rev 79:211–227. doi: 10.1177/0003122413519445.Are
19. Turanovic JJ, Pratt TC (2015) Longitudinal effects of violent victimization during adolescence on adverse outcomes in adulthood: A focus on prosocial attachments. J Pediatr 166:1062–1069.e1. doi: 10.1016/j.jpeds.2014.12.059
20. Kidd S, Henrich C (2006) The social context of adolescent suicide attempts: Interactive effects of parent, peer, and school social relations. Suicide Life-Threatening Behav 36:387–395. doi: 10.1521/suli.2006.36.4.386
21. Rodríguez-Cano T, Beato-Fernández L, Llario AB (2006) Body dissatisfaction as a predictor of self-reported suicide attempts in adolescents: A Spanish community prospective study. J Adolesc Heal 38:684–688. doi: 10.1016/j.jadohealth.2005.08.003
22. Ackard D, Eisenberg M, Neumark-Sztainer D (2007) Long-term impact of adolescent dating violence on the behavioral and psychological health of male and female youth. J Pediatr 151:476–481
23. Brezo J, Paris J, Barker ED, et al (2007) Natural history of suicidal behaviors in a population-based sample of young adults. Psychol Med 37:1563–1574. doi: 10.1017/S003329170700058X
24. Brezo J, Paris J, Vitaro F, et al (2008) Predicting suicide attempts in young adults with histories of childhood abuse. Br J Psychiatry 193:134–139. doi: 10.1192/bjp.bp.107.037994
25. Crow S, Eisenberg ME, Story M, Neumar-Sztainer D (2008) Are Body Dissatisfaction, Eating Disturbance and Body Mass Index Predictors of Suicidal Behavior in Adolescents? A Longitudinal Study. J Consult Clin Psychol 76:887–892. doi: 10.1037/a0012783.Are
26. Dupéré V, Leventhal T, Lacourse É (2009) Neighborhood poverty and suicidal thoughts and attempts in late adolescence. Psychol Med 39:1295–1306. doi: 10.1017/S003329170800456X
27. Lambert SF, Copeland-Linder N, Ialongo NS (2008) Longitudinal Associations Between Community Violence Exposure and Suicidality. J Adolesc Heal 43:380–386. doi: 10.1016/j.jadohealth.2008.02.015
28. Nrugham L, Larsson B, Sund AM (2008) Predictors of suicidal acts across adolescence: influences of familial, peer and individual factors. J Affect Disord 109:35–45. doi: 10.1016/j.jad.2007.11.001
29. Nrugham L, Holen A, Sund AM (2015) Prognosis and psychosocial outcomes of attempted suicide by early adolescence: a 6-year follow-up of school students into early adulthood. J Nerv Ment Dis 203:294–301. doi: 10.1097/NMD.0000000000000281
30. Wong JPS, Stewart SM, Claassen C, et al (2008) Repeat suicide attempts in Hong Kong community adolescents. Soc Sci Med 66:232–241. doi: 10.1016/j.socscimed.2007.08.031
31. Wilcox HC, Storr CL, Breslau N (2009) Posttraumatic Stress Disorder and Suicide Attempts in a Community Sample of Urban American Young Adults. Arch Gen Psychiatry 66:305–311
32. Batty GD, Whitley E, Kivimäki M, et al (2010) Body mass index and attempted suicide: Cohort study of 1,133,019 Swedish men. Am J Epidemiol 172:890–899. doi: 10.1093/aje/kwq274
33. Peter T, Roberts LW (2010) “Bad” boys and “Sad” girls? examining internalizing and externalizing effects on parasuicides among youth. J Youth Adolesc 39:495–503. doi: 10.1007/s10964-009-9498-5
34. Roberts RE, Roberts CR, Xing Y (2010) One-year incidence of suicide attempts and associated risk and protective factors among adolescents. Arch Suicide Res 14:66–78. doi: 10.1080/13811110903479078
35. Klomek A, Kleinman M, Altschuler E, et al (2012) High school Bullying as a Risk for Later Depression and Suicidality. Suicide Life-Threatening Behav 41:501–516. doi: 10.1111/j.1943-278X.2011.00046.x.High
36. Young R, Sweeting H, Ellaway A (2011) Do schools differ in suicide risk? the influence of school and neighbourhood on attempted suicide, suicidal ideation and self-harm among secondary school pupils. BMC Public Health 11:874. doi: 10.1186/1471-2458-11-874
37. Fried LE, Williams S, Cabral H, Hacker K (2012) Differences in Risk Factors for Suicide Attempts Among 9th and 11th Grade Youth: A Longitudinal Perspective. J Sch Nurs 29:113–122. doi: 10.1177/1059840512461010
38. Guan K, Fox KR, Prinstein MJ (2012) Nonsuicidal Self-Injury as a Time-Invariant Predictor of Adolescent Suicide Ideation and Attempts in a Diverse Community Sample. J Consult Clin Psychol 80:842–849. doi: 10.1037/a0029429.Nonsuicidal
39. Hurtig T, Taanila A, Moilanen I, et al (2012) Suicidal and self-harm behaviour associated with adolescent attention deficit hyperactivity disorder-a study in the Northern Finland Birth Cohort 1986. Nord J Psychiatry 66:320–8. doi: 10.3109/08039488.2011.644806
40. Nkansah-Amankra S, Diedhiou A, Agbanu SK, et al (2012) A longitudinal evaluation of religiosity and psychosocial determinants of suicidal behaviors among a population-based sample in the United States. J Affect Disord 139:40–51. doi: 10.1016/j.jad.2011.12.027
41. Wanner B, Vitaro F, Tremblay RE, Turecki G (2012) Childhood trajectories of anxiousness and disruptiveness explain the association between early-life adversity and attempted suicide. Psychol Med 42:2373–82. doi: 10.1017/S0033291712000438
42. Winterrowd E, Canetto SS (2013) The long-lasting impact of adolescents’ deviant friends on suicidality: a 3-year follow-up perspective. Soc Psychiatry Psychiatr Epidemiol 48:245–255. doi: 10.1007/s00127-012-0529-2
43. Chang S Sen, Chen YY, Heron J, et al (2014) IQ and adolescent self-harm behaviours in the ALSPAC birth cohort. J Affect Disord 152–154:175–182. doi: 10.1016/j.jad.2013.09.005
44. Chen C-Y, Yeh HH, Huang N, Lin YC (2014) Socioeconomic and clinical characteristics associated with repeat suicide attempts among young people. J Adolesc Heal 54:550–557. doi: 10.1016/j.jadohealth.2013.10.008
45. Conner KR, Bossarte RM, Lu N, et al (2014) Parent and Child Psychopathology and Suicide Attempts among Children of Parents with Alcohol Use Disorder Kenneth. Arch suicide Res 18:117–130. doi: 10.1080/13811118.2013.826154.Parent
46. Luntamo T, Sourander A, Gyllenberg D, et al (2014) Do headache and abdominal pain in childhood predict suicides and severe suicide attempts? Finnish Nationwide 1981 Birth Cohort study. Child Psychiatry Hum Dev 45:110–118. doi: 10.1007/s10578-013-0382-x
47. Mars B, Heron J, Crane C, et al (2014) Differences in risk factors for self-harm with and without suicidal intent: Findings from the ALSPAC cohort. J Affect Disord 168:407–414. doi: 10.1016/j.jad.2014.07.009
48. Miranda R, Ortin A, Scott M, Shaffer D (2014) Characteristics of suicidal ideation that predict the transition to future suicide attempts in adolescents. J Child Psychol Psychiatry 55:1288–1296. doi: 10.1111/jcpp.12245
49. Soller B (2014) Caught in a Bad Romance. J Health Soc Behav 55:56–72. doi: 10.1177/0022146513520432
50. Swanson EN, Owens EB, Hinshaw SP (2014) Pathways to self-harmful behaviors in young women with and without ADHD: A longitudinal examination of mediating factors. J Child Psychol Psychiatry Allied Discip 55:505–515. doi: 10.1111/jcpp.12193
51. Scott LN, Pilkonis PA, Hipwell AE, et al (2015) Non-suicidal self-injury and suicidal ideation as predictors of suicide attempts in adolescent girls: A multi-wave prospective study. Compr Psychiatry 58:1–10. doi: 10.1016/j.comppsych.2014.12.011
52. You J, Lin MP (2015) Predicting suicide attempts by time-varying frequency of nonsuicidal self-injury among Chinese community adolescents. J Consult Clin Psychol 83:524–533. doi: 10.1037/a0039055
53. Conway PM, Erlangsen A, Teasdale TW, et al (2016) Predictive Validity of the Columbia-Suicide Severity Rating Scale for Short-Term Suicidal Behavior: A Danish Study of Adolescents at a High Risk of Suicide. Arch Suicide Res 21:455–469. doi: 10.1080/13811118.2016.1222318
54. Meza JI, Owens EB, Hinshaw SP (2016) Response Inhibition, Peer Preference and Victimization, and Self-Harm: Longitudinal Associations in Young Adult Women with and without ADHD. J Abnorm Child Psychol 44:323–334. doi: 10.1007/s10802-015-0036-5
55. Mok PLH, Pedersen CB, Springate D, et al (2016) Parental psychiatric disease and risks of attempted suicide and violent criminal offending in offspring a population-based cohort study. JAMA Psychiatry 73:1015–1022. doi: 10.1001/jamapsychiatry.2016.1728
56. Hishinuma ES, Smith MD, McCarthy K, et al (2017) Longitudinal Prediction of Suicide Attempts for a Diverse Adolescent Sample of Native Hawaiians, Pacific Peoples, and Asian Americans. Arch Suicide Res 22:67–90. doi: 10.1080/13811118.2016.1275992
57. King CA, Raskin A, Gdowski CL, et al (1990) Psychosocial Factors Associated with Urban Adolescent Female Suicide Attempts. J Am Acad Child Adolesc Psychiatry 29:289–294. doi: 10.1097/00004583-199003000-00020
58. Rotheram-Borus MJ, Trautman PD, Dopkins SC, Shrout PE (1990) Cognitive style and pleasant activities among female adolescent suicide attempters. J Community Health 58:554–561
59. Garnefski N, Diekstra RF, de Heus P (1992) A population-based survey of the characteristics of high school students with and without a history of suicidal behavior. Acta Psychiatr Scand 86:189–196. doi: 10.1111/j.1600-0447.1992.tb03250.x
60. Adams DM, Overholser JC, Spirito A (1994) Stressful life events associated with adolescent suicide attempts. Can J Psychiatry 39:43–48
61. Beautrais AL, Joyce PR, Mulder RT (1998) Psychiatric illness in a New Zealand sample of young people making serious suicide attempts. N Z Med J 111:44–48
62. Lyon ME, Benoit M, O´Donell RM, et al (2000) Assessing african american adolscents risk for suicide attempts: Adolescence 35:122–134
63. Ikeda RM, Kresnow MJ, Mercy J a, et al (2001) Medical conditions and nearly lethal suicide attempts. Suicide Life-Threatening Behav 32:60–7. doi: 10.1521/suli.32.1.5.60.24207
64. Donald M, Dower J, Correa-Velez I, Jones M (2006) Risk and protective factors for medically serious suicide attempts: A comparison of hospital-based with population-based samples of young adults. Aust N Z J Psychiatry 40:87–96. doi: 10.1111/j.1440-1614.2006.01747.x
65. Bilgin M, Cenkseven F, Satar S (2007) An analysis of parent-female adolescent relationships in female adolescent suicides. Crisis 28:190–197. doi: 10.1027/0227-5910.28.4.190
66. Freitas GVS, Cais CFS, Stefanello S, Botega NJ (2008) Psychosocial conditions and suicidal behavior in pregnant teenagers: A case-control study in Brazil. Eur Child Adolesc Psychiatry 17:336–342. doi: 10.1007/s00787-007-0668-2
67. Christiansen E, Goldney RD, Beautrai a L, Agerbo E (2011) Youth suicide attempts and the dose-response relationship to parental risk factors: a population-based study. Psychol Med 41:313–319. doi: 10.1017/S0033291710000747
68. Christiansen E, Stenager E (2012) Risk for attempted suicide in children and youths after contact with somatic hospitals: a Danish register based nested case-control study. J Epidemiol Community Heal 66:247–253. doi: 10.1136/jech.2009.103887
69. Finkelstein Y, Macdonald EM, Hollands S, et al (2015) Long-term outcomes following self-poisoning in adolescents: A population-based cohort study. The Lancet Psychiatry 2:532–39. doi: 10.1016/S2215-0366(15)00170-4
70. Feigelman W, Joiner T, Rosen Z, Silva C (2016) Investigating Correlates of Suicide Among Male Youth: Questioning the Close Affinity Between Suicide Attempts and Deaths. Suicide Life-Threatening Behav 46:191–205. doi: 10.1111/sltb.12183
71. Weiser M, Fenchel D, Werbeloff N, et al (2017) The association between premorbid cognitive ability and social functioning and suicide among young men: A historical-prospective cohort study. Eur Neuropsychopharmacol 27:1–7. doi: 10.1016/j.euroneuro.2016.11.015
72. Salk L, Sturner W, Lipsitt L, et al (1985) Relationship of maternal and perinatal conditions to eventual adolescent suicide. Lancet 325:624–627. doi: 10.1016/S0140-6736(85)92156-7
73. Brent DA, Perper JA, Moritz G, et al (1993) Firearms and Adolescent Suicide A Community Case-Control Study. Am J Dis Child 147:1066–1071
74. Brent DA, Baugher M, Bridge J, et al (1999) Age- and Sex-Related risk factors for adolscents suicide. J Am Acad Child Adolesc Psychiatry 38:1497–1505
75. Shaffer D, Gould MS, Fisher P, et al (1996) Psychiatric Diagnosis in Child and Adolescent Suicide. Arch Gen Psychiatry 55:339–348
76. Cheng C-CJ, Yen W-J, Chang W-T, et al (2014) Risk of adolescent offspring’s completed suicide increases with prior history of their same-sex parents’ death by suicide. Psychol Med 44:1845–1854. doi: 10.1017/S0033291713002298
77. Ostry A, Maggi S, Tansey J, et al (2007) The impact of psychosocial work conditions on attempted and completed suicide among western Canadian sawmill workers. Scand J Public Health 35:265–71. doi: 10.1080/14034940601048091

**FIGURE S1.** Galbraith plot (a) and Funnel plot (b) of being female as risk factor of suicide attempt – results of the

systematic review of gender differences in suicidal behavior in adolescents and young adults (covered up until January 2017)


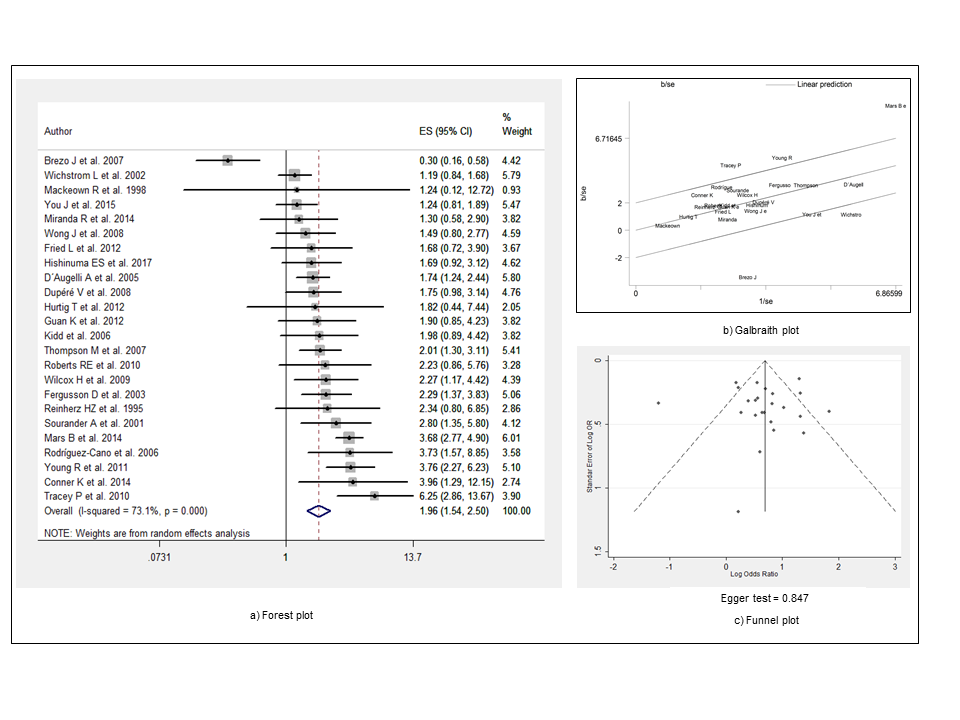

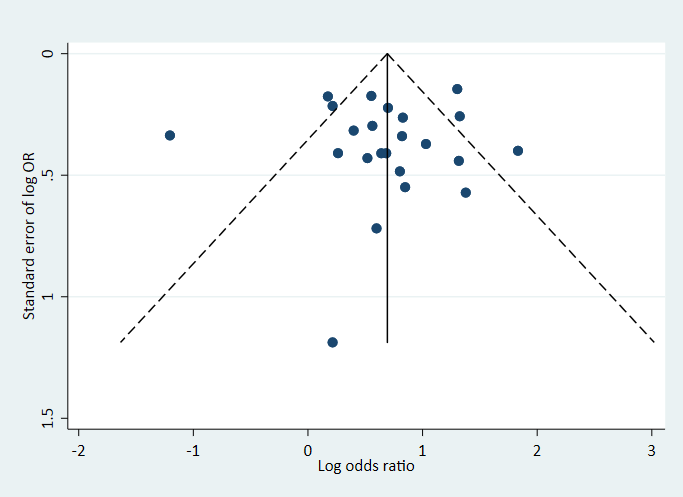


**(a) Galbraith plot (b) Funnel plot**

Egger test = 0.847
